# Thermal Conductivity of Carbon/Boron Nitride Heteronanotube and Boron Nitride Nanotube Buckypapers: Implications for Thermal Management Composites

**DOI:** 10.1021/acsanm.3c01147

**Published:** 2023-06-22

**Authors:** Ruth Sang Jones, Sergio Gonzalez-Munoz, Ian Griffiths, Philip Holdway, Koen Evers, Santamon Luanwuthi, Barbara M. Maciejewska, Oleg Kolosov, Nicole Grobert

**Affiliations:** † University of Oxford, Department of Materials, Oxford OX1 3PH, United Kingdom; ‡ University of Lancaster, Department of Physics, Lancaster LA1 4YB, United Kingdom; ¶ Williams Advanced Engineering, Grove, Oxfordshire OX12 0DQ, United Kingdom

**Keywords:** boron nitride nanotubes, carbon nanotubes, heteronanotubes, buckypapers, thermal management, scanning thermal microscopy

## Abstract

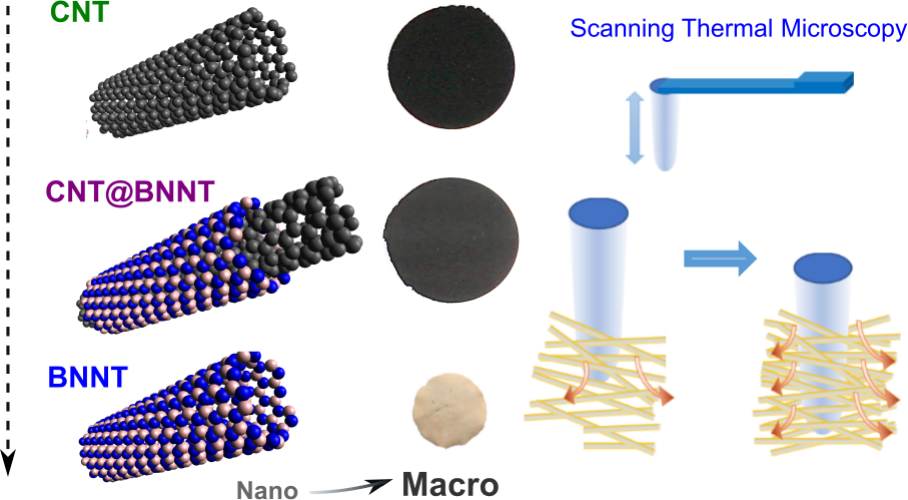

To date, there has been limited reporting on the fabrication and properties of macroscopic sheet assemblies (specifically buckypapers) composed of carbon/boron nitride core–shell heteronanotubes (MWCNT@BNNT) or boron nitride nanotubes (BNNTs). Herein we report the synthesis of MWCNT@BNNTs via a facile method involving Atmospheric Pressure Chemical Vapor Deposition (APCVD) and the safe h-BN precursor ammonia borane. These MWCNT@BNNTs were used as sacrificial templates for BNNT synthesis by thermal oxidation of the core carbon. Buckypaper fabrication was facilitated by facile sonication and filtration steps. To test the thermal conductivity properties of these new buckypapers, in the interest of thermal management applications, we have developed a novel technique of advanced scanning thermal microscopy (SThM) that we call piercing SThM (pSThM). Our measurements show a 14% increase in thermal conductivity of the MWCNT@BNNT buckypaper relative to a control multiwalled carbon nanotube (MWCNT) buckypaper. Meanwhile, our BNNT buckypaper exhibited approximately half the thermal conductivity of the MWCNT control, which we attribute to the turbostratic quality of our BNNTs. To the best of our knowledge, this work achieves the first thermal conductivity measurement of a MWCNT@BNNT buckypaper and of a BNNT buckypaper composed of BNNTs not synthesized by high energy techniques.

## Introduction

The term buckypaper was first coined in 1998 in reference to a “free-standing mat of entangled SWCNTs”, as SWCNTs of “molecular perfection” belong to the fullerene family of carbon allotropes^1^ and are sometimes called buckytubes. Nowadays, the term is used more broadly to encompass all carbon nanotube (CNT, both single-walled (SWCNT) and multiwalled (MWCNT)) paper-like assemblies^2,3^ and has also been co-opted for similar architectures of non-carbon nanotube materials, e.g., boron nitride nanotube (BNNT) buckypapers. Regardless of the type of nanotube, the similar feature shared by all buckypapers is that they are held together by the van der Waals forces acting between nanotubes tangled into a mesh-like, porous network. The intrinsic properties along the nanotube axis, such as a high thermal conductivity (e.g., 300 W m^–1^ K^–1^ of CNTs and of 350 W m^–1^ K^–1^ BNNTs),^4^ can be leveraged at the macroscopic scale by the formation of interconnected pathways in the buckypaper network.

Buckypapers, by nature of being a macroscopic assembly, are easier to handle than loose nanotube powders. This is particularly relevant to nanocomposite manufacturing, e.g. nanotubes embedded in a polymer matrix. When mixed with a liquid matrix during composite processing, powder nanotube additives have a tendency to agglomerate, while also increasing the viscosity of the mixture.^5^ These factors can contribute to nonuniform distribution of the nanotubes in the matrix as well as a limited achievable nanotube content due to poor processability of highly viscous mixtures.^6^ Buckypapers offer a competitive alternative path to nanocomposite fabrication. Their pre-existing nanotube network is porous and can be impregnated with the matrix material, thus allowing a high content of nanotubes that are already homogeneously distributed to become part of the composite.^7^ For example, BNNT polymer/epoxy composites with the highest nanotube load thus far attained (17–40 wt %) were fabricated by infiltration of the matrix into the preformed buckypaper.^8−11^ These higher nanotube loads are promising for a more weighted contribution of nanotube properties to the overall composite, e.g., greater improvements in thermal conductivity.^8−10^

Besides being lightweight, buckypapers also present the advantages of being paper-like thin sheets that are usually flexible. These features make it a practical material for fashioning into a desired geometry or bending/folding to fit a form, otherwise not always feasible with other macroassemblies such as aerogels/foams^12^ or aligned carpets.^13^

To date, the research output on the fabrication of nanotube buckypapers is still largely CNT focused, which is a consequence of maturation of the field to facilitate scaled synthesis of CNTs. Meanwhile, the synthesis and investigation of BNNT buckypapers is still at a relatively nascent stage, in part due to challenges of synthesis and scalability of production of BNNTs. The same can be said for heteronanotube buckypapers. A summary of BNNT and heteronanotube sheet-like assemblies that have been synthesized to date is provided in Table S2 of the Supporting Information. Among these studies, there has been one report of a mat type assembly composed of compressed BCN heteronanotubes.^14^ More recently hybrid buckypapers were fabricated with intermixed CNTs and BNNTs,^15^ or separate layers of BNNT and CNTs. For example, sandwich buckypapers of a BNNT core layer with CNT outer layers^9^ or a CNT core layer insulated by BNNT layers^11^ have been fabricated, as well as so-called Janus, two sided BNNT/CNT buckypapers.^9^ There has been a very limited variety of the BNNT dimensions in these reported buckypapers (usually <10 nm diameter with 2–5 walls) owing to the BNNTs being fabricated by the same highly specialized HABS^16^ or HTP^17^ methods, which also tend to produce other BN polymorphs that lower the purity of the BNNT product.

BNNTs present certain advantages over their CNT counterparts. Unlike CNTs that are metallic or semiconducting dependent on their chirality,^18^ BNNTs are consistently electrically insulating.^19^ This property, coupled with their superb elastic modulus, makes BNNTs the strongest known insulating fiber to date.^20^ They also possess the incredibly rare duo of being a dielectric and a thermal conductor. BNNTs also outperform CNTs’ thermal stability and can resist oxidation in air up to up to 800–900 °C^21−23^ (and up to 1500 °C in inert atmosphere).^9^ Other BNNT properties such as demonstration of a piezoelectric effect^24,25^ and high neutron adsorption capacity (due to the large neutron adsorption cross section of boron^26^) are not presented by CNTs.

As we recently highlighted in our review,^27^ many of the advantageous properties of h-BN, such as a higher thermal and chemical stability, can be conferred to CNTs by the formation of coaxial heteronanotubes with a core CNT and outer shell BNNT, i.e., a CNT@BNNT structure. The h-BN layers add further pathways for thermal conduction along the nanotube while providing a dielectric barrier to electrically insulate the core conductive CNTs.

In view of the favorable thermal and dielectric properties of both BNNTs and MWCNT@BNNTs, they are both promising candidates for use in novel heat dissipating materials, e.g., in polymer composites for passive thermal management in electronics packaging and next generation flexible electronics.^5,7−10,28,29^

**Figure 1:**
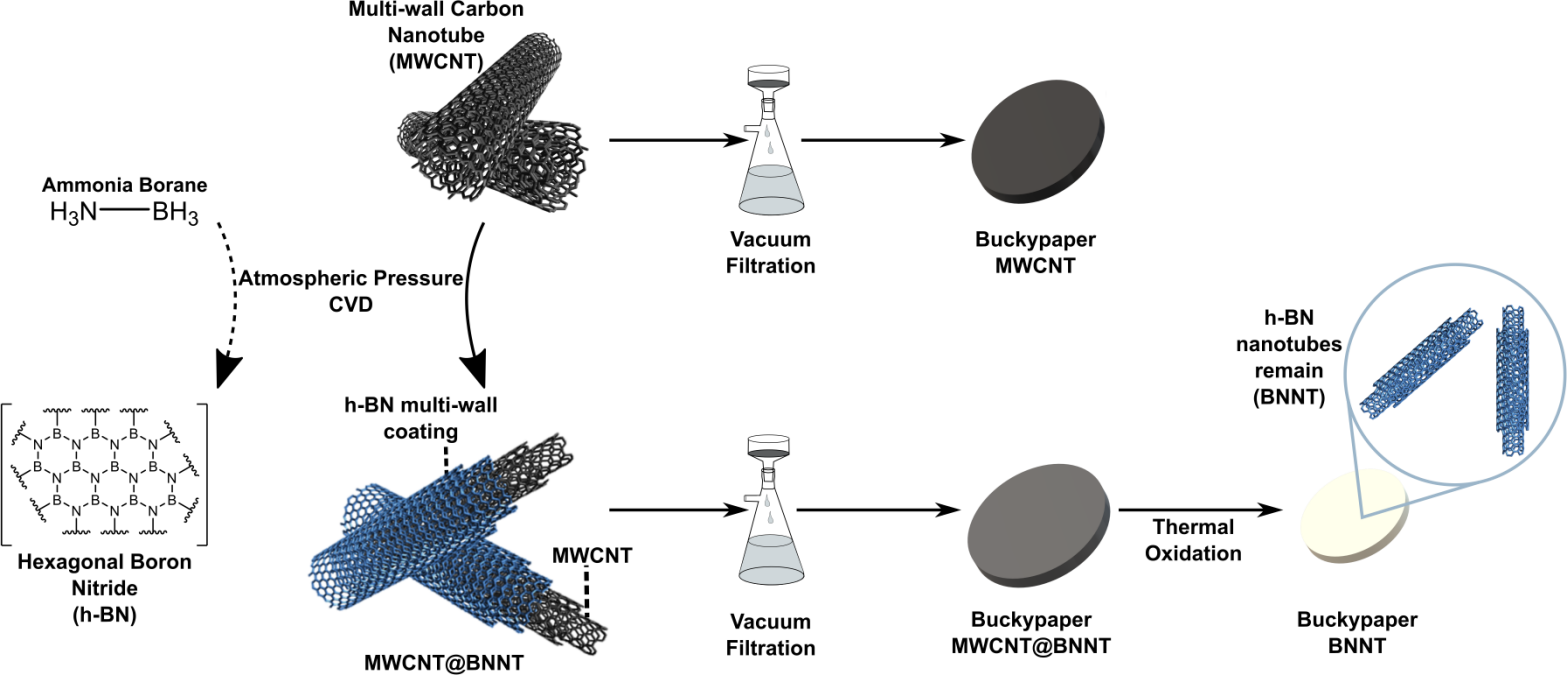
Schematic illustrating the steps of fabricating the various nanotube species and their associated macroscopic buckypaper assemblies in this work.

In this paper, we report the fabrication of MWCNT@BNNT and BNNT and their respective buckypapers by using a combination of Atmospheric Pressure Chemical Vapor Deposition (APCVD), thermal oxidation, and vacuum filtration, as shown in the Figure 1 schematic. We further measure buckypaper thermal conductivity with a novel method of advanced scanning thermal microscopy (SThM).

Our APCVD method is economic and technically feasible for scaled production and involves the use of a single source precursor ammonia borane, which is a particularly viable precursor for the CVD synthesis of h-BN. It is a stable solid at room temperature (safe for storage) and is also nonhazardous (easy to handle). Therein lies one of its advantages over many other precursors such as boron trihalides, diborane, and borazine which themselves present risks associated with being toxic, highly flammable, or corrosive or produce harmful byproducts.^30^

## Results and Discussion

**Figure 2:**
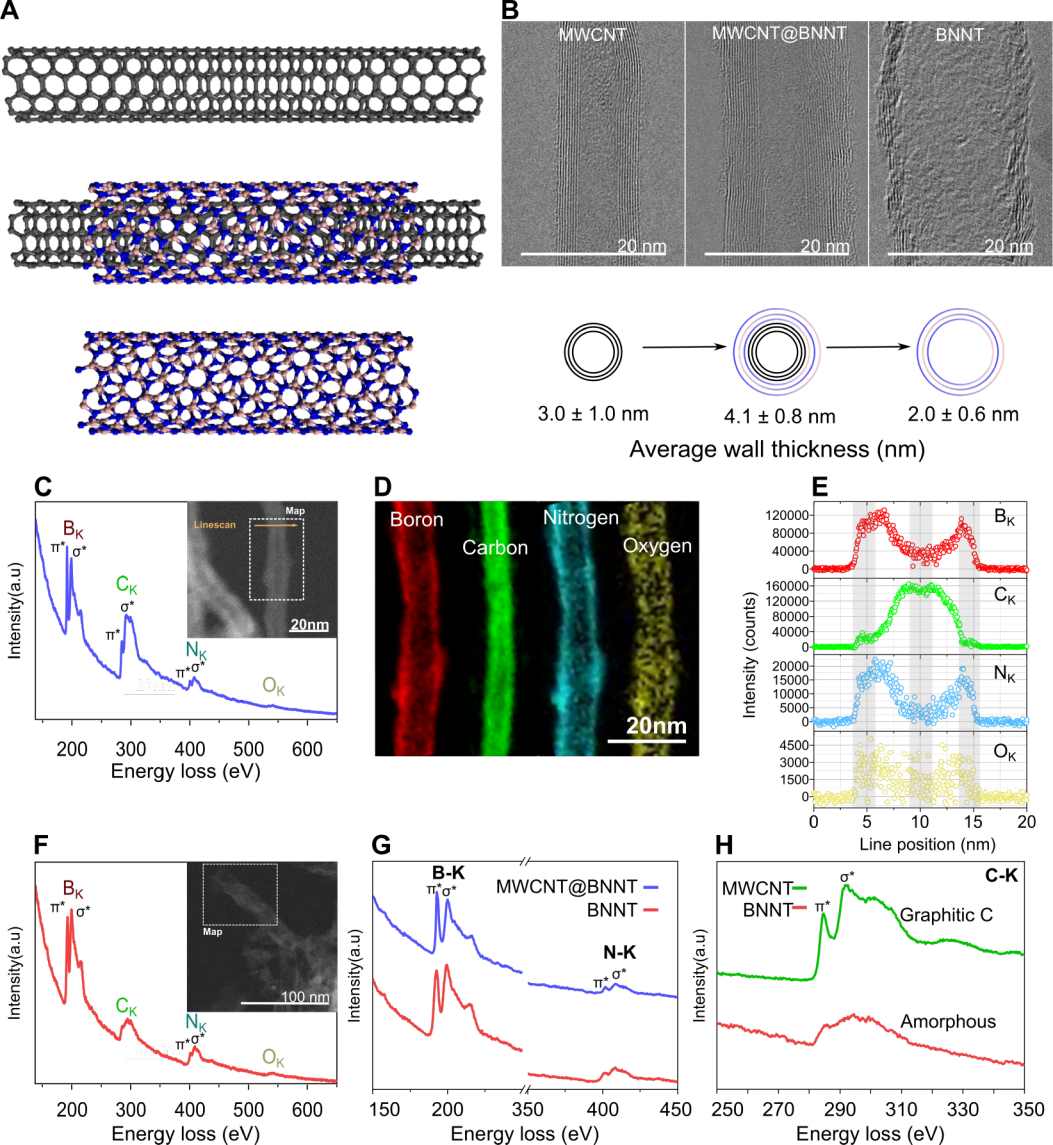
(A) Schematic representation of nanotubes in this work, transforming from a CNT, to a heteronanotube of BN coated on the CNT, to a BNNT. Only single and double-wall nanotubes are shown for simplicity.(B) HRTEM images of the transformation of MWCNTs to MWNCNT@BNNT to BNNT achieved in this work. Average wall thicknesses of each nanotube species are summarized in the accompanying illustration. (C) EELS spectrum of MWCNT@BNNT as mapped from inset STEM image, showing B K, C K, N K and O K edges. (D) EELS spectral images of indicated mapped region in panel C, confirming a BN shell around a carbon core. (E) EELS line scan conducted at indicated lateral position along the MWCNT@BNNT. The profile shows distinct BN rich outer layers around a graphitic core. (F) EELS spectrum of BNNT as mapped from inset STEM image, showing B K, N K, and O K edges and a reduced C K edge. (G) Comparison of B K and N K edges of MWCNT@BNNT and BNNT indicating similar h-BN structure. (H) Comparison of C K edges of the BNNT and MWCNT profiles; the BNNT C K edge is unlike that of graphitic carbon and is most similar to amorphous carbon.

The synthesized MWCNT@BNNT and BNNT structures were investigated by electron microscopy characterization, including SEM, TEM, and EELS. An increased outer diameter of the MWCNT@BNNTs relative to the pristine MWCNT, as measured from HRTEM images, reveals the introduction of an h-BN outer coating with a average thickness of 2 ± 0.6 nm (Figure 2B). Subsequent to oxidation, the wall thickness of the residual BNNT corresponds to the thickness of these deposited h-BN layers. Additional EELS maps (Figure S7) revealed evidence of the h-BN coating also encapsulating the tip of MWCNTs. This tip encapsulation is further corroborated by the observation of close-ended BNNTs (Figure S5).

The observed lamellar nature of the deposited coating confirms its h-BN polymorph structure, further evidenced by the collected EELS high-loss spectra, which depict B K and N K edges with π* and σ* fine features. The appearance of π* and σ* peaks in graphite and h-BN core-loss ELNES (Energy Loss Near Edge Structures) can be interpreted according to valence-bond theory of their sp^2^ bond hybridization, alongside molecular orbital theory on antibonding orbitals. The sp^2^ hybrid orbitals between C atoms (or B–N atoms) in-plane leads to σ bonds while unhybridized p orbitals perpendicular to the internuclear axis form delocalized π bonds. Empty antibonding σ* and π* orbitals are the energy states to which excited 1s core electrons are promoted, and the energy associated with this transition is manifested in the ELNES of the C K ionization edge of graphite or the B K and N K edges of h-BN. These fine features are present in both the EELS spectra of the MWCNT@BNNTs (Figure 2C) and the BNNTs (Figure 2F). EELS spectral maps and line scans clearly depict the colocation of B and N in an outer shell encapsulating a core MWCNT (Figure 2D,E).The oxygen profile, albeit with its lower relative intensity, can be roughly superposed onto the matching B and N profiles, suggesting that oxygen is also present at the h-BN outer walls. The source of this oxygen is discussed later on.

While the hollow nature of the BNNTs is evident from TEM images (e.g., Figure 2B), this is not as easily visible from the STEM image of a BNNT presented in Figure 2F. This is a result of issues such as sample charging and drifting during the EELS mapping, which negatively affected the achieved spatial resolution. It must thus be acknowledged that future work is needed to optimize the EELS collection parameters for these thin-wall, insulating BNNT samples. Nevertheless, the preliminary EELS analysis of these BNNTs confirms that B and N are present and remain in their sp^2^ bonded networks expected of h-BN, based on the π* and σ* features of B K and N K edge profiles that are very similar to those of the MWCNT@BNNT precursor, as compared in Figure 2G. Interestingly, the presence of carbon could indicate the incomplete removal of the MWCNT cores during sacrificial templating. However, further inspection of the C K edge profile of the BNNTs in comparison to that of graphitic MWCNT (Figure 2H) reveals that C K edge of the BNNTs closely resembles that of amorphous carbon,^16,31^ with noticeably weaker π* and σ* features than apparent on the C K edge of graphitic carbon. Such amorphous carbon is theorized to originate from adventitious carbon contamination, as previously demonstrated in other BNNT products in the literature,^16^ and which is prevalent on all surfaces exposed to air.^32^ Theoretical studies have also predicted that defect sites in BNNTs are highly reactive sites for CO_2_ chemisorption.^33^ An alternative hypothesis is that the carbon may be residual from the MWCNT but no longer graphitic due to oxidative damage and bonded to the BNNTs in such a way to avoid volatilization during the oxidation process. Given that there is limited evidence of covalent bonding between the h-BN and MWCNT components, as summarized below, the adventitious carbon hypothesis is preferred here.

**Figure 3:**
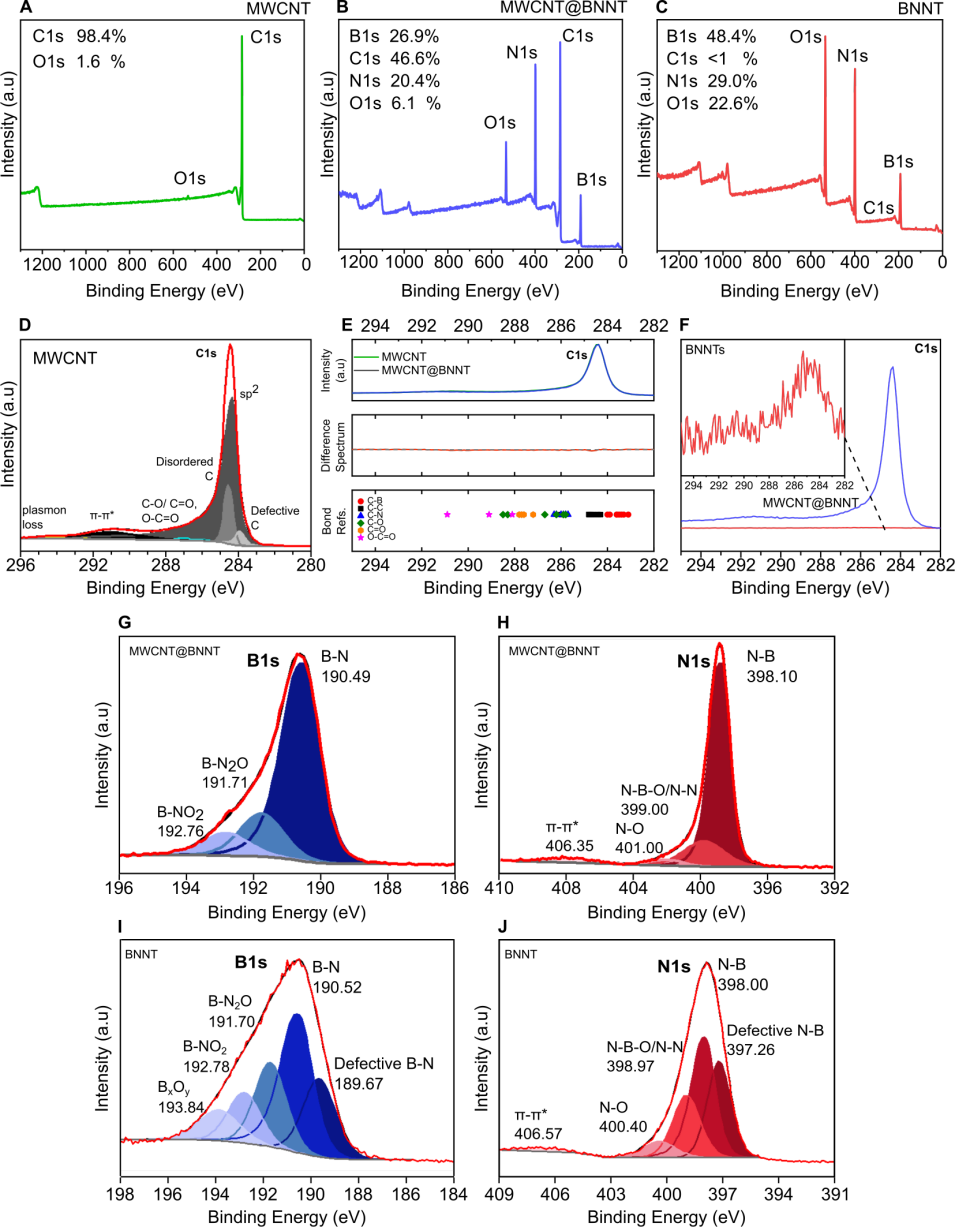
XPS survey spectra of (A) Nanocyl NC7000 MWCNTs, (B) MWCNT@BNNTs, and (C) BNNTs. (D) XPS high resolution C 1s spectra of Nanocyl NC7000 MWCNT. (E) Superposed high resolution XPS C 1s spectra of Nanocyl NC7000 MWCNTs and MWCNT@BNNTs. The difference spectrum shows no noticeable peaks in the C–B or C–N regions, as compared to spectra in the literature,^13,29,34−43^ suggesting few covalent interactions between the MWCNT core and h-BN shell. (F) High resolution C 1s spectrum of BNNTs normalized to the C 1s spectrum of the MWCNT@BNNTs. The low intensity C 1s spectrum of the BNNTs is magnified in the inset. High resolution (G) B 1s spectrum and (H) N 1s spectrum of MWCNT@BNNTs. High resolution (I) B 1s spectrum and (J) N 1s spectrum of BNNTs. See Supporting Information Tables S4–S6 for further details on peak fitting of all spectra.

XPS provides further evidence in support of the core–shell structure of the synthesized MWCNT@BNNTs and the subsequent extent of graphitic carbon removal from the BNNTs. The change in composition of the different nanotube species is shown in progression in the XPS survey spectra of Figure 3A–C. High resolution C 1s, B 1s, and N 1s XPS spectra of the MWCNT@BNNTs suggest that the MWCNT and h-BN components are ordered in core–shell layers interacting by van der Waals forces. Particularly, the MWCNTs C 1s spectrum, shown deconvoluted in Figure 3D, is almost identical to the MWCNT@BNNTs C 1s spectrum. When the two C 1s spectra are superposed, their difference spectrum is calculable, as shown in Figure 3E. This difference spectrum shows no noticeable peaks in the C–B or C–N regions, as compared to spectra in the literature,^13,29,34−43^ suggesting that the core graphitic lattice remains largely intact and without appreciable covalent bonding with B/N. This is corroborated by corresponding B 1s and N 1s spectra which reveal that B and N are predominantly bonded to each other in a h-BN lattice (Figure 3G,H). This conclusion is further supported by UV–vis spectroscopy (Figure S10) and Raman spectroscopy (Figure S13).

Subsequently, XPS of the BNNTs reveals residual carbon content <1 atom %, with the C 1s spectrum exhibiting a peak characteristic of amorphous carbon (Figure 3F). Broadening of the B 1s XPS spectrum to higher binding energies also points to an increase in B–O related bonds in the BNNT structure (Figure 3I), even including free oxide B_*x*_O_*y*_ (e.g., B_2_O_3_).^44−46^ This points to the partial oxidation of the BNNTs, as corroborated by FTIR (Figure S12).

As observed from EELS of the MWCNT@BNNTs (Figure 2C–E) and the high resolution XPS B 1s spectrum (Figure 3G), oxygen functionalities are present and are distributed within the h-BN coating, primarily in B–O covalent bonds such as B–N_2_O and B–NO_2_.^47^ There may be multiple sources of this oxygen contamination, including in the quartz tube reactor during CVD and post-CVD cooling, and/or out of the quartz tube reactor, i.e., during sample storage. Considering that the implemented APCVD system is always open to air in intermission periods between uses and that there is no complex vacuum pumping regime prior to use, it is likely, even described as inevitable,^48^ that residual atmospheric gases are present in the system after purging. This is a compromise made for the time and cost-saving simplicity of the APCVD system. Hence, oxygen-containing atmospheric gases, e.g., residual O_2_ and/or H_2_O, could be present within the CVD reactor during use. However, measures in the CVD method were also in place to counteract the effects of oxidizing impurities. Specifically, 2.5% H_2_ was present in the carrier gas alongside inert Ar. H_2_ has the supplementary role of reducing oxidizing impurities within an APCVD system, such that the potential for these impurities to “oxidatively etch” vulnerable materials in the system is minimized.^48^ Alternatively, oxygen contamination occurs during exposure of the MWCNT@BNNTs to ambient atmosphere during storage, which is a theory supported by literature on the phenomenon of hydrolytic attack of h-BN by ambient moisture.^49−55^ Specifically, h-BN hydrolytic stability has a direct relationship with h-BN crystallinity; as the crystallinity of h-BN decreases, synonymous with increasing *d*_002_ interlayer spacing and the density of defects, moisture sensitivity also increases.^49,56^ Accordingly, ambient H_2_O, initially physisorbed onto the h-BN could interact with h-BN primarily at reactive defect sites such as dangling bonds to covalently incorporate oxygen, e.g., by bonded hydroxyl groups.^54,55^

As characterized by TEM, both the MWCNT core and deposited h-BN exhibit imperfect crystallinity. As anticipated, the walls of the Nanocyl NC7000 MWCNTs have a turbostratic ordering that limits the consequent ordering of the deposited h-BN.^27,57^ Thus, in the context of h-BN moisture sensitivity being determined by its crystallinity, the h-BN deposited in this work could be unstable under ambient conditions and susceptible to reaction with H_2_O. A further consequence of the disordered, turbostratic nature of the deposited h-BN is its lower resistance to thermal oxidation, thereby explaining the partial oxidation of the BNNTs after oxidation at 700 °C while highly crystalline h-BN can resist oxidation beyond 900 °C (Figure S11). Such oxidation is likely to occur at the h-BN defect sites, and a higher density of defects after oxidation may further contribute to the instability of the BNNTs to ambient moisture, therefore contributing to the rise in oxygen in the BNNTs to 22.6% compared to the 6.1% in the MWCNT@BNNTs according to XPS (Figure 3B,C). This increase in oxygen content in the BNNTs is accompanied by a change in the B/N atomic ratio from 1.32:1 for MWCNT@BNNTs to 1.67:1 for BNNTs, which is an inflated bias toward B. This is in keeping with the preferential substitution of O at N vacancy sites instead of B sites because of a more similar electronegativity of O with N.^47,58^ Further, N oxidation product, N_2_, is more easily volatilized than B oxidation product, B_2_O_3_.^56,59−61^

**Figure 4:**
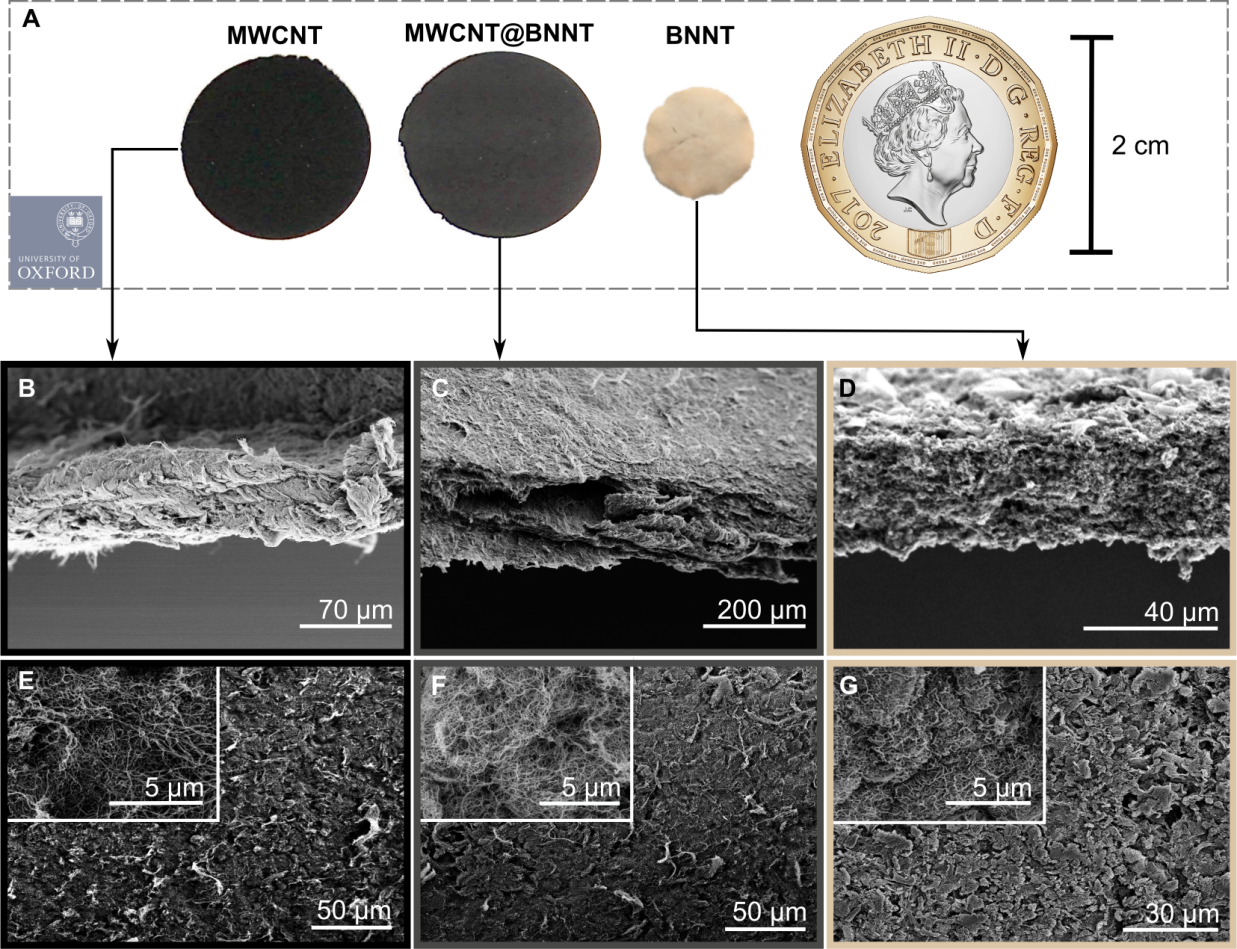
(A) Optical images of MWCNT, MWCNT@BNNT, and BNNT buckypapers as fabricated herein and shown to scale. (B, C) Cross sectional views and (E–G) surface views of the corresponding buckypapers.

Randomly entangled networks of the MWCNT@BNNTs and BNNTs were fabricated that resulted in self-supporting sheets of buckypaper at the macroscopic scale, as shown in Figure 4. Buckypaper thicknesses achieved averaged 115–140 μm for the MWCNT@BNNTs and 80–90 μm for the BNNTs. Generally, the assemblies fabricated are low density architectures, with bulk densities in the range of approximately 0.12–0.2 g/cm^3^. Owing to the process of fabrication of these buckypapers, which involves a step of vacuum filtration, the buckypapers possess a layered structure, with better *xy* plane alignment within these layers compared to along the buckypaper *z*-axis, most evident in Figure 4C. Upon handling, the MWCNT@BNNT buckypaper (like the MWCNT control) was found to be flexible while the BNNT buckypaper was brittle. One factor that may contribute to this change is a decreased length of the nanotubes subsequent to the thermal oxidation procedure, resulting in reduced entanglement.

Of particular interest for the application of these buckypapers (BPs) is their thermal conductivity. However, their extremely soft and porous nature provides a major challenge for measurement of thermal conductivity by methods such as the time domain reflectance^62^ or 3-omega^63^ which require deposition of additional structures on the BP, thereby affecting the thermal conductivity. Advanced scanning thermal microscopy (SThM) methods that use a nanoscale sized tip in contact with the sample such as cross-sectional SThM^64^ or SThM of the individual nanofibers^65^ would be a better choice, but a different approach is required given that there is no sharp tip–sample contact in case of BPs.

We therefore developed a new SThM approach that we call “piercing SThM”, or pSThM, that measures the heat conductance into the sample as a function of penetration of the probe tip Δ*Z* into the buckypaper. The thermal conductance into the BP is *g*_BP_, which is given by *g*_BP_ = *q*_BP_/Δ*T*, where Δ*T* is the temperature difference between the tip and the sample and *q*_BP_ is the heat flow into the sample. The thermal conductance *g*_BP_ is also proportional to the thermal conductivity of the BP, *k*_BP_, and the area of contact *A* between the tip and the sample, which is given as *A* = *L*Δ*Z*, where *L* is an averaged perimeter of the probe that can be approximated, at small indentation distances, as a cylindrical surface. A thermal conductance into the BP sample, *g*_BP_ (the value measured in the SThM), is then expressed as *g*_BP_ = α*L*Δ*Zk*_BP_, where α is a coefficient with dimension of m^–1^ and on the order of inverse diameter of the probe,^66^ with α being the same for all BPs that we are comparing.

**Figure 5:**
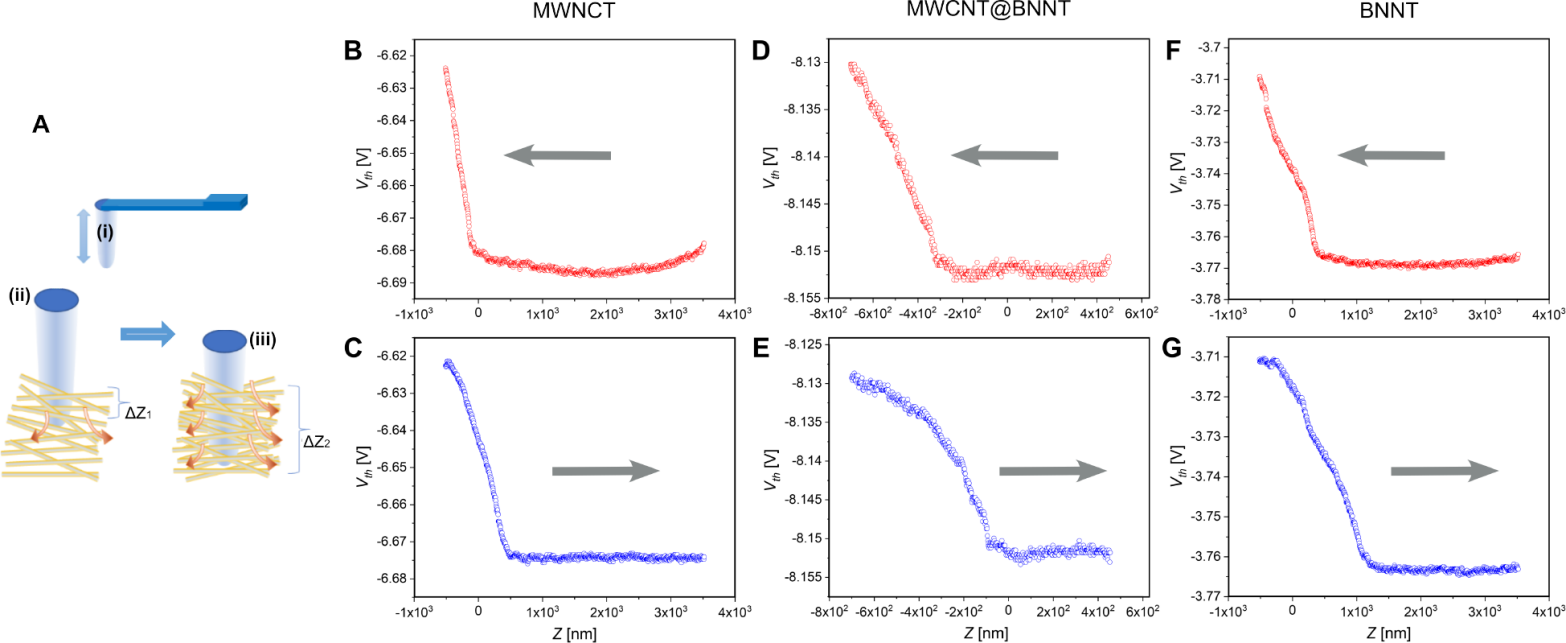
(A) Heat transfer from the scanning thermal microscope (SThM) tip (i) increases as the tip “punctures” the BP sample with penetration depth into the BP layer increasing from Δ*Z*_1_ (ii) to Δ*Z*_2_ (iii). The heat flow into the sample, *q*_BP_, is proportional to the contact area *L* × Δ*Z* where *L* is the perimeter of the probe. Plots show dependencies of the probe “thermal signal” for penetration (“approach”; B, D, F) and withdrawal (“retract”; C, E, G) of the probe to and from the BP layer for MWCNT-BP (B, C), MWCNT@BNNT-BP (D, E), and BNNT-BP (F, G). The thermal signal is linearly proportional to the decrease of the probe temperature in contact with the BP layer allowing one to directly calculate the heat conductance of the probe–sample contact.

The results of pSThM measurements indeed indicate the absence of the initial tip–surface “jump” of the thermal signal, that would be typical for the solid samples.^67^ Approximately linear initial increase of the “thermal signal” in Figure 5B,D,F reflects the linear increase of the heat flow into the sample as expected for the pSThM tip “puncturing” the BP sample as illustrated in Figure 5A. It is useful to note that both the “puncture” (approach) and withdrawal (retract) produce similar dependencies, with retract expected to extend over larger distance as BP fibers gradually release the retracting SThM tip.

Due to the extremely soft nature of the BPs and the relatively stiff SThM probe we used, the deflection of the tip was negligible compared to the displacement of the sample, allowing use of the sample displacement Δ*Z* as the penetration depth (Figure 5A). The “thermal signal” can be converted into the tip–sample thermal conductance (see Methods), *g*_BP_ = *g*_0_δ*T*/Δ*T* = *αL*Δ*Zk*_BP_. The derivative of this value per piercing depth is given by , and so the experimentally measured ratio δ*T*/(Δ*Z*Δ*T*) is then directly proportional to the thermal conductivity of the BP sample, *k*_BP_.

**Figure 6:**
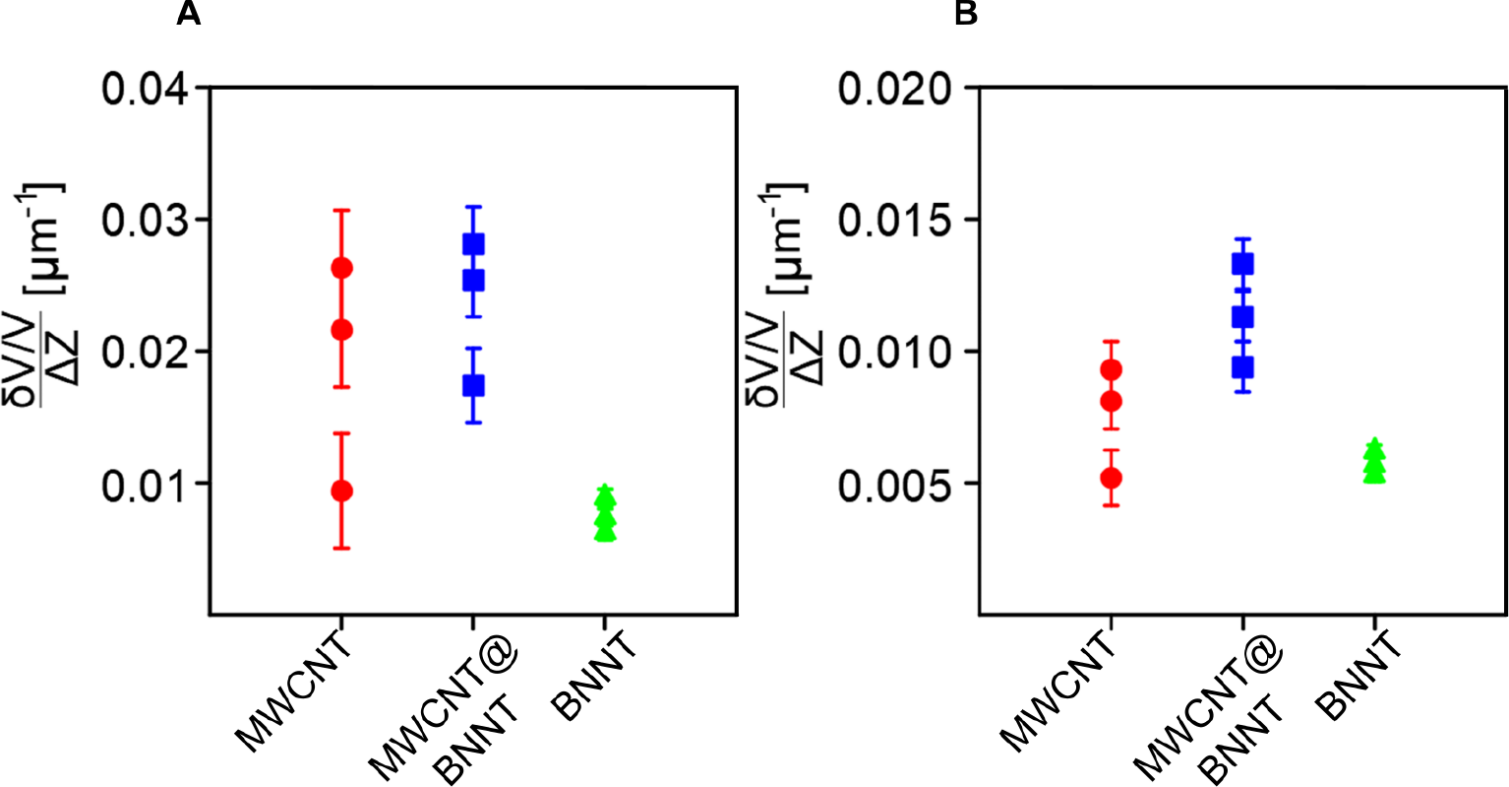
pSThM technique measures the ratio given by the derivative of the relative change of the probe temperature per increment of penetration depth δ*T*/(Δ*Z*Δ*T*). The probe voltage equivalent of this derivative is used as the *Y*-axis unit. This ratio is directly proportional to the thermal conductivity of each of the BPs.

The plots in Figure 6 compare the values of the derivative δ*T*/(Δ*Z*Δ*T*) for three samples (MWCNT-BP, BNNT-BP and MWCNT@BNNT-BP) indicating that while BNNT-BP has the lowest thermal conductivity of approximately 30% of MWCNT-BP and MWCNT@BNNT-BP, the combined MWCNT@BNNT-BP has superior conductivity of all three samples. It is possible to provide approximate estimates of the thermal conductivity (κ) of the samples, assuming a good thermal contact between the tip and BP and that the perimeter *L* of the SPM tip is 2π of the radius. Then substituting the (δ*g*_BP_)/Δ*Z* values for each BP, we obtain 1.64 ± 0.5 W m^–1^ K^–1^ for MWCNT-BP, 1.87 ± 0.5 W m^–1^ K^–1^ for MWCNT@BNNT-BP and 0.76 ± 0.2 W m^–1^ K^–1^ for BNNT-BP. For reference, a typical polymer has a (κ) in the range of 0.15–0.3 W m^–1^ K^–1^. Considering that values in the literature of the κ of sheet assemblies of MWCNTs is typically in the range of 1–10 W m^–1^ K^–1^,^68^ our approximations here by pSThM for the κ of the MWCNT-BP fall within this anticipated range and so improve the confidence in the κ values measured for the MWCNT@BNNT-BP and the BNNT-BP. Further, because the BPs are porous, there is space among the nanotubes that effectively make the BPs a heterogeneous material. Although the pSThM measurements were conducted in vacuum and so there is no air in the BPs, the empty spaces still contribute toward the measured thermal conductivity. As such, quoted κ values are in fact for the apparent or effective thermal conductivity (κ_app_).

Notably, however, these BP (κ) values are still magnitudes lower than that of individual nanotubes. This is largely due to the thermal contact resistance at nanotube–nanotube junction sites as well as nanotube misalignment,^69^ both of which are areas for improvement to be targeted by future studies.

According to our estimations, the MWCNT@BNNT-BP exhibits a (κ) increase of 14% relative to the pristine MWCNT-BP, indicating the effectiveness of the coaxial BNNT outer layers at introducing additional thermal conductance pathways along the nanotubes. This is accompanied by a 5-fold increase in the electrical resistivity of the MWCNT@BNNT-BP to ca. 0.5 Ω·cm compared to the ca. 0.1 Ω·cm resistivity of the MWCNT-BP, which is attributable to h-BN acting as a dielectric barrier at the junctions between nanotubes in the buckypaper network (Figure S16). The MWCNT@BNNT-BP also benefits from improved thermal stability relative to the MWCNT-BP (see thermogravimetric analysis in Figure S11).

On the other hand, while the BNNT-BP also exhibits a higher κ than typical polymer, its estimated value is still the lowest of the three BP varieties. This is likely attributable to the partial oxidation, reduced crystallinity, and likely reduced length of the BNNTs subsequent to oxidation treatment to remove the CNT core. These factors will affect the κ of each individual nanotube, and accordingly the conductivity of the bulk assembly.^10,70^ To our knowledge, investigation of BNNT-BP κ is as of yet very scarce. The κ of a compressed a BNNT pellet composed of 10–80 nm BNNTs synthesized by a catalytic reaction of Mg and B_2_O_2_ was reported as 0.96 W m^–1^ K^–1^.^71^ Using hydrogen assisted BNNT synthesis (HABS), in situ diaphanous sheets and vacuum filtered BPs have been fabricated with effective κ in the range of 0.5–0.8 W m^–1^ K^–1^ and 1.3–1.7 W m^–1^ K^–1^ respectively.^10^ By comparison, our approximated BNNT-BP κ is on the lower end of these reported ranges, but nevertheless at the same order of magnitude.

## Conclusion

Using a scalable and accessible APCVD method implementing the safe h-BN precursor ammonia borane, we synthesized MWCNT@BNNTs by coating MWCNT templates with ca. 2 nm of coaxial h-BN layers. Subjecting the MWCNT@BNNTs to thermal oxidation resulted in sacrificing of the MWCNTs to leave residual BNNT shells. We also demonstrate assembly of these MWCNT@BNNTs and BNNTs at the macroscopic scale in the form of buckypapers and report the first thermal conductivity approximations of these buckypapers using a novel pSThM technique.

Our thermal conductivity approximations suggest a positive effect of introducing coaxial h-BN onto the surface of the MWCNTs, such that the MWCNT@BNNT-BP had a 14% improved thermal conductivity relative to that of a pure MWCNT-BP. Coupled with an increased electrical resistivity compared to the MWCNT-BP, we demonstrate that the properties of the MWCNT@BNNT-BP make it better suited than the pure MWCNT-BP for incorporation into an electrically insulating yet thermally conductive composite.

Meanwhile, our BNNT-BP exhibits a thermal conductivity lower than both the MWCNT-BP and the MWCNT@BNNT-BP. We mainly attribute this to the low crystallinity of the BNNTs and their oxygen impurities, which is a result of BNNT partial oxidation. There is potential for future studies to address the issue of improving the crystallinity of the BNNTs. Nevertheless, the unique turbostraticity of these BNNTs may find uses in other applications besides thermal management, such as for CO_2_ and/or H_2_O gas adsorption at the BNNT defect sites.

Finally, in comparison to BNNTs synthesized by other CVD techniques, the BNNTs synthesized here have the advantage of being highly selective to cylindrical nanotube morphologies, i.e., they are structurally homogeneous and have no other h-BN polymorph impurities. While other CVD fabricated BNNTs tend to have larger outer diameters, >100 nm, owing to size dependency on thermoagglomerated catalysts, and high energy techniques tend to produce BNNTs with small diameters, <10 nm, this method facilitates tunable BNNT diameters based solely on the diameters of the starting MWCNT template. Consequently, it is envisaged that this method will allow fabrication of a broad range of BNNT buckypapers with a diversity of BNNT dimensions, which has yet to be achieved.

## Methods

### Synthesis of Boron Nitride Coated Multiwall Carbon Nanotubes (MWCNT@BNNT)

Ammonia borane powder was mixed with inert h-BN filler in the weight ratio of 1:10 (100 mg and 1000 mg) to separate the ammonia borane granules and prevent global polymerization of volatile gases emitted upon heating. This precursor mixture was transferred into a reservoir flask that was connected upstream to the horizontal quartz tube furnace.

Nanocyl NC7000 MWCNTs (2–3 mg) were spread flat to form a roughly 2 × 2 cm square at the center of a cleaned quartz slide (Figure S2). This slide was then inserted into the quartz tube at a room temperature position, and the system was sealed and purged with Ar/H_2_ (2.5%) for 25 min.

To begin the synthesis (see Figure S1), an electronically heated jacket was raised to envelop the precursor reservoir flask. This cover was preheated to a set temperature of 90 °C before synthesis was initiated. Once the precursor flask began heating, the furnace-on-wheels was shifted over the region of growth, ensuring that the preheated 1000 °C center of the furnace aligned with the center of the growth sample. The connecting stainless steel manifold between the reservoir and the reactor quartz tube was also heated to 100 °C to prevent gas condensation inside the connector. Synthesis was conducted for 2 h in Ar/H_2_ (2.5%) net carrier gas flow of 400 sccm. Gas flow through the precursor flask was set to 100 sccm. After this set time, the furnace was shifted downstream, allowing the sample to cool to room temperature within 15 min in Ar/H_2_ (2.5%) flow. The system was then unsealed, and the powder sample was then retrieved and stored in a glass vial.

Prior to use of the reactants and reactant vessels, decontamination procedures were conducted as summarized here. Ammonia borane and h-BN powder were vacuum treated for 5 days prior to use. This treatment was introduced to remove any residual organic solvent, such as tetrahydrofuran (THF) in the 97% grade Sigma-Aldrich product. Such extensive vacuum treatment has been previously found to be effective at carbon decontamination.^72^ Nanocyl MWCNT powder was also vacuum treated and stored in a desiccator prior to use to ensure the absence of residual solvents and moisture. The support apparatus for containing the powder nanotubes (quartz slide) was cleaned by solvent rinsing (in order: acetone, methanol, isopropanol), followed by drying with the nitrogen gun. This support apparatus was heat treated inside the quartz tube growth environment up to 1000 °C in air flow to oxidize any carbon contaminants present on the supports or in the reactor prior to the experiment. Glass precursor reservoir flasks were also cleaned following the same solvent wash, and subjected to UV/ozone treatment for 30 min prior to each experimental run.

### Synthesis of MWCNT and MWCNT@BNNT Buckypapers

For fabricating MWCNT buckypapers, MWCNT nanotube powder ca. 4 mg was dispersed into 20 mL of ethanol solution in a glass vial. This solution was then sonicated in an ultrasonic bath at room temperature for 4 h at a frequency of 20 kHz. Following sonication, the dispersion was vacuum filtered onto Whatman’s Cyclopore track etched, polycarbonate filter paper with 0.4 μm pore size. The buckypaper and filter paper sample was vacuum treated overnight, and then the buckypaper was carefully peeled off the filter paper.

The same procedure was conducted for fabrication of MWCNT@BNNT buckypaper, using MWCNT@BNNT powder (as synthesized according to the method above) as the starting material.

Due to the diameter of the filtration cylinder in contact with the filter paper, all buckypapers synthesized were 17 mm in diameter.

### Synthesis of BNNT Buckypaper

BNNT buckypaper was fabricated by a sacrificial templating method. Accordingly, a MWCNT@BNNT buckypaper (as synthesized by the method described above) was positioned horizontally on a quartz slide, which was placed into the quartz reactor tube inside the furnace (same setup used for CVD in Figure S1). The buckypaper was subsequently oxidized under air flow (1.8 L/min from an air pump) at 700 °C for 1 h for selective removal of graphitic components while leaving residual h-BN in the buckypaper.

### Measurement of Thermal Conductance of BP Layers Using a Scanning Thermal Microscope (SThM)

We have used a vacuum SThM (NT-MDT Solver) operating at 10^–4^ Torr range to eliminate spurious thermal transport through the air^67^ and a Si SThM probe (VITA-HE-NANOTA-200, Bruker) with spring constant of 2 N/m. The probe formed part of the balanced Wheatstone bridge that was excited by the 400 mV 91 kHz AC voltage and 600 mV DC voltage creating approximately 40 K excess temperature with respect to the BP sample. The output “thermal signal” at 91 kHz proportional to the probe resistance and, hence, its temperature, was measured via the lock-in amplifier (SRS-830, Stanford Research Systems) at the 3–10 ms time constant.^73^ The temperature of the probe Δ*T* (with respect to the temperature of the sample) is found as Δ*T* = *W*_p_/*g*_0_ where *W*_p_ is the power applied to the probe and *g*_0_ is the heat conductance of the probe in the absence of the sample. Contact with the BP sample adds the conductance *g*_BP_ to *g*_0_ resulting in the probe temperature drop to Δ*T* – δ*T* and the new relation Δ*T* – δ*T* = *W*_p_/(*g*_0_ + *g*_BP_). Accounting that δ*T* ≪ Δ*T* and that W_p_ is constant,^73^ we can find the thermal conductance of the sample as *g*_BP_ = *g*_0_δ*T*/Δ*T* with *g*_0_ = 1.87 × 10^–5^ W K^–1^.

## Supporting Information Available

Additional experimental details, including schematics of experimental setups, and additional characterization data (PDF)
