# Supplementary material for: Thermal Conductivity of Carbon/Boron Nitride Heteronanotube and Boron Nitride Nanotube Buckypapers: Implications for Thermal Management Composites
Source: ACS Appl Nano Mater. 2023 Jun 22;6(17):15374–84. doi: 10.1021/acsanm.3c01147 (PMC10496026; doi:10.1021/acsanm.3c01147)
Supplement: Supplementary file 1 — an3c01147_si_001.pdf [file an3c01147_si_001.pdf]

Supporting Information: Thermal Conductivity of Carbon/Boron Nitride Heteronanotube and Boron Nitride Nanotube Buckypapers: Implications for Thermal Management Composites

Ruth Sang Jones,† Sergio Gonzalez-Munoz,‡ Ian Griffiths,† Philip Holdway,† Koen Evers,† Santamon Luanwuthi,† Barbara M. Maciejewska,*,† Oleg Kolosov,‡ and Nicole Grobert*,†,¶

†*University of Oxford, Department of Materials, Oxford, OX1 3PH, United Kingdom*

‡*University of Lancaster, Department of Physics, Lancaster, LA1 4YB, United Kingdom*

¶*Williams Advanced Engineering, Grove, Oxfordshire, OX12 0DQ, United Kingdom*

E-mail: barbara.maciejewska@materials.ox.ac.uk; nicole.grobert@materials.ox.ac.uk

Synthesis of Boron Nitride coated Multiwall Carbon Nanotubes (MWCNT@BNNT)

Table S1: Summary of reactants implemented in this method for synthesis of MWCNT@BNNT, and solvents used for cleaning of substrates, sample holders *etc.*

**Name**	**Purity**	**Supplier**
	**Reactants**	
Solids		
MWCNT Nanocyl NC7000™	90%	Nanocyl SA
Ammonia Borane Complex	97%	Sigma-Aldrich
h-BN powder grade 015	>98.5%	3M
Gases		
Argon/Hydrogen (97.5 : 2.5 %)	-	BOC
	**Solvents**	
Acetone		Sigma Aldrich
Isopropanol		Sigma Aldrich
Methanol		Sigma Aldrich

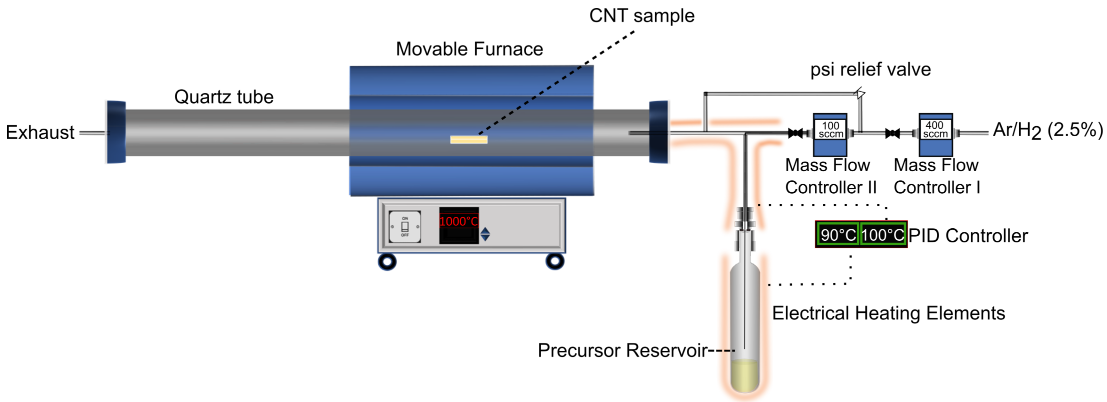

Figure S1: Illustration of CVD setup for h-BN depoisition onto MWCNTs by Ammonia Borane pyrolysis. Furnace: Elite Model TSU/12/38/500. Quartz tube dimensions: Length =1.8 m, inner diameter=29 mm .

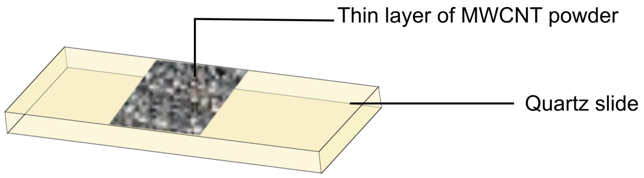

Figure S2: Illustration of CNT sample setup inside the reactor, i.e. layer of CNT on quartz slide.

Table S2: Summary of BNNT , BCNT and CNT+BNNT macroscopic sheet assemblies reported in the literature, sorted according to the method of fabrication. The dimensions of nanotubes in the assemblies are compiled, along with the macroscopic assemblies' processing steps and overall thickness and density.

		BNNT characteristics			Processing Steps	Assembly Characteristics		
**Types of BNNT Freestanding Sheet Macroassemblies**	**Nanotube synthesis method**	**Diameter (nm)**	**No. of walls**	**Length (μm)**		**Thickness (μm)**	**Density (g/cm^3)**	**Ref**
In Situ Growth Techniques								
**a.** BNNT cloth-like deposits	HABS	ca. 5	2-5	Few	Formed in HABS reactor	Few mm (thin film layers peeled off e.g. 0.2 μm)	0.15	[1],[2]
**b.** Partially aligned BNNT Buckypaper	HABS	ca. 5	2-5	Few	Deposited on rotating cylinder inside HABS reactor	–	0.16	[3]
**c.** Bamboo BNNT thin film	N-ISC+CVD	60-80	>30	–	Substrate spraycoated w/ boron+catalyst ink; Heat treated in N_2 +H_2, Peeled off with blade	20-50	0.12	[4]
Filtration Techniques								
**d.** BNNT Buckypaper, BNNT+CNT hybrid Buckypaper (Sandwich, Janus)	HABS	ca. 5	2-5	Few	Purification,Stirring, Horn/Bath Sonication Filtration (Water/Methanol)	15-150	0.3-0.5	[1],[2] [3],[5]
**e.** BNNT Buckypaper, Sandwich Buckypaper (SWCNT/BNNT/SWCNT)	HTP	1-10	2-5	ca. 200	Sonication, Filtration (N-methyl-pyrrolidone)	ca. 10 (in sandwich)	–	[6]
**f.** BNNT films, thicker mats	HTP	1-10	2-5	ca. 200	Mixing, Centrifugation Supernatant Extraction Filtration (Chlorosulfonic Acid)	<1-1.5 & thicker mat	–	[7]
Compression Techniques								
**g.** BCNNT mats, BNNT mats	Substitution/ Oxidation	6-8	<10	–	BCNNTs Pressed, Annealed, Oxidized	700	–	[8]
**h.** Cylinder BNNT pellet,<br>Bamboo BNNT pellet,<br>Collapsed BNNT pellet	BOCVD<br>BBO+NH_3 CVD<br>BOCVD+Pt catalysis	10-80<br>10-100<br>10-80	ca. 10<br>>100<br>-	ca. 10<br>–<br>ca. 10	Pressed with 40 MPa	1000-2000	–	[9]
**i.** BNNT mat	HTP	1-10	2-5	ca. 200	Pressed with 5.8 kPa	2300-6300	0.02	[10]
**j.** BNNT mat	HTP	1-10	2-5	ca. 200	Pressed 0.47-22.97 MPa	145-706	0.11-0.51	[11]
This Work								
**k.** MWCNT@BNNT Buckypaper	APCVD	10-20	ca. 9-15	–	Bath Sonication (Ethanol), Filtration	115-140	0.1-0.2	–
**l.** BNNT Buckypaper	APCVD/ Oxidation	10-20	ca. 4-8	–	Oxidation of MWCNT@BNNT BP	80-90	0.1-0.2	–

Additional characterisation data

SEM and TEM

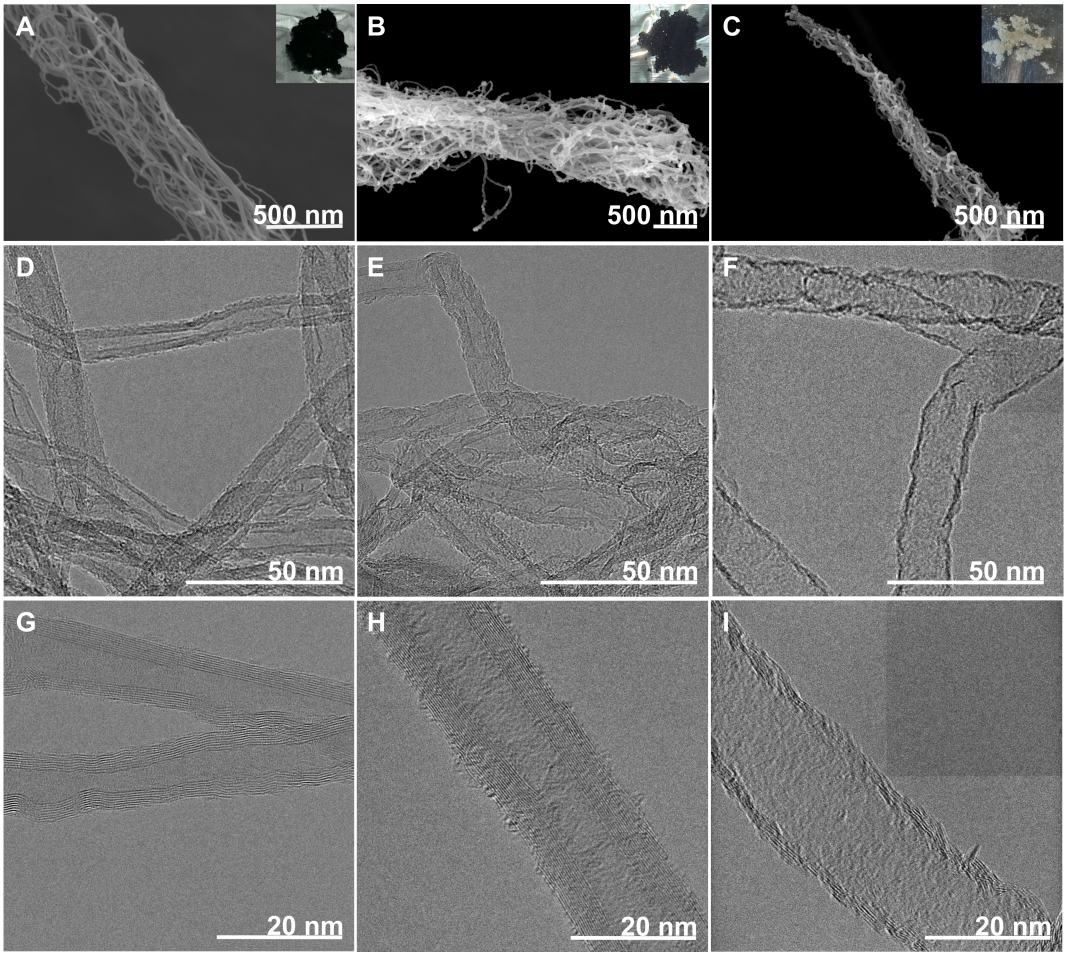

Figure S3: (A-C) SEM images of MWCNT, MWCNT@BNNT and BNNTs respectively (Inset camera images of loose powder nanotubes). (D-E) Additional TEM images of MWCNTs, MWCNT@BNNT and BNNTs respectively. (G-I) Additional high magnification TEM images of MWCNT, MWCNT@BNNTs and BNNTs respectively.

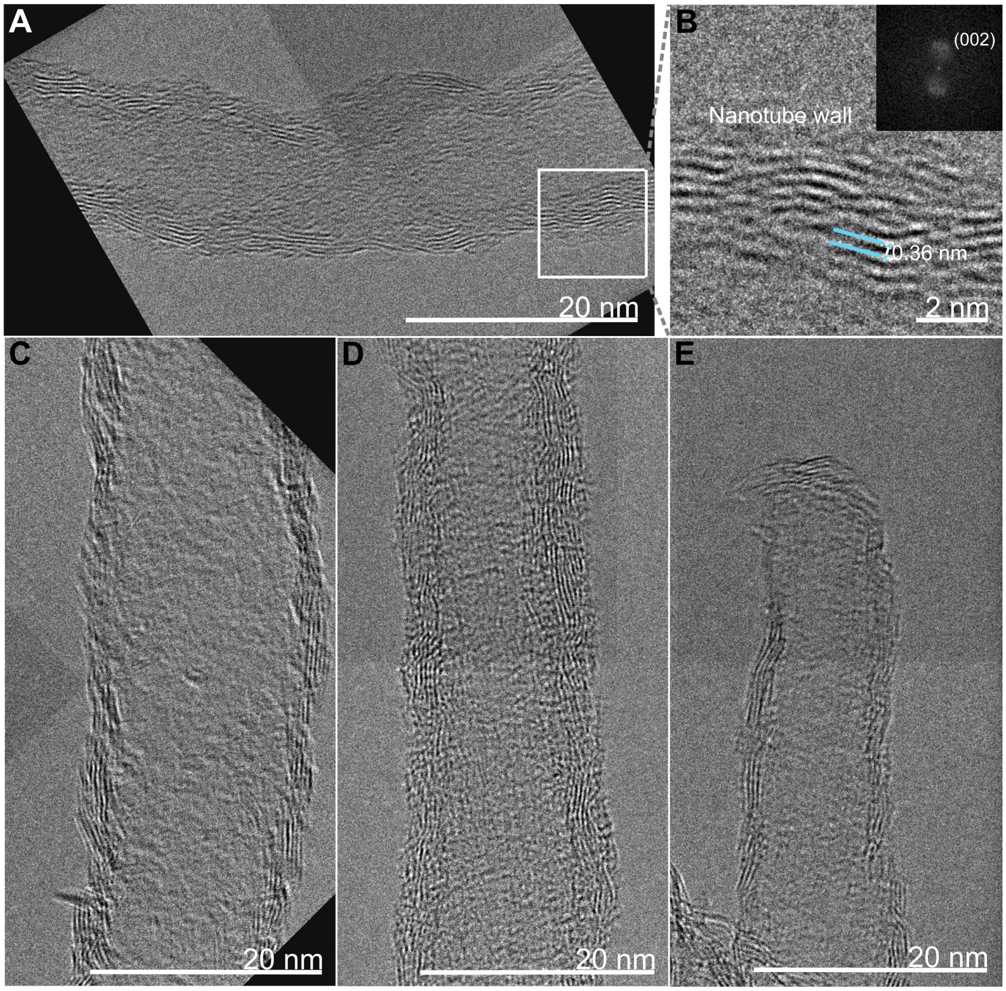

Figure S4: Example HRTEM images of BNNTs synthesised here by sacrificial templating. These images evidence the disordered structure of the BNNT walls. The achieved turbostratic BNNT wall structure is magnified in (B), showing the disordered h-BN lamellae and a broad (002) spacing of ca. 0.36 nm.

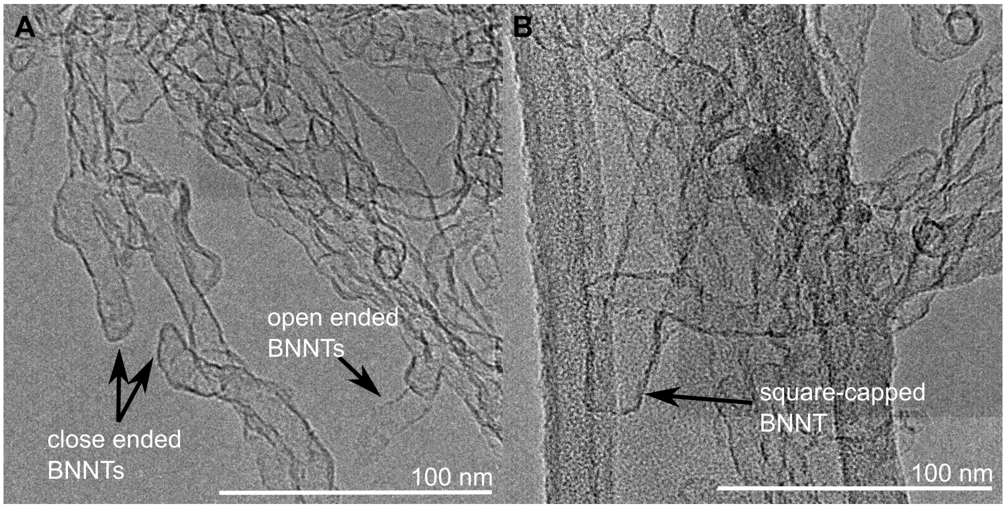

Figure S5: TEM images depicting BNNTs with different tip structures, e.g. closed vs open ended.

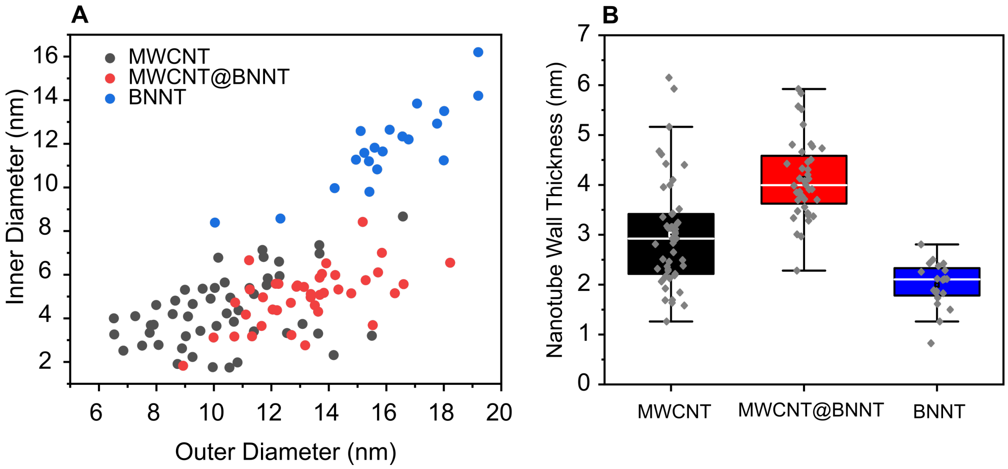

Figure S6: (A) Diameter spread and (B) Wall thickness distributions of MWCNT, MWCNT@BNNT and BNNTs as measured from HRTEM images.

Electron Energy Loss Spectroscopy

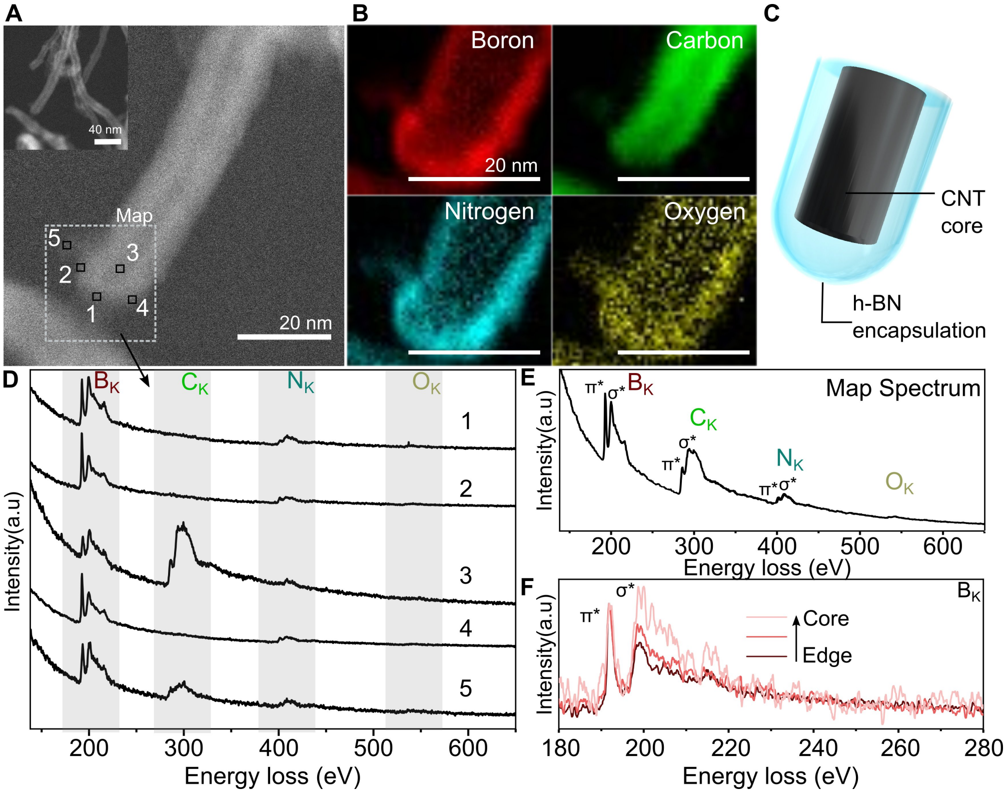

Figure S7: MWCNT@BN specimen observed by (A)Dark Field STEM with the nanotube tip mapped by (B) EELS spectral imaging. Complete tip encapsulation by BN is illustrated in (C). EELS spectra are extracted from the coordinates labelled 1-5 (D), indicating the h-BN composition of the outer shell. An atypical appendage is observed at coordinate 5. Increasing intensity of B-K edge σ^* peak relative to π^* (outer shell to inner core) indicates 360° h-BN encapsulation (F).

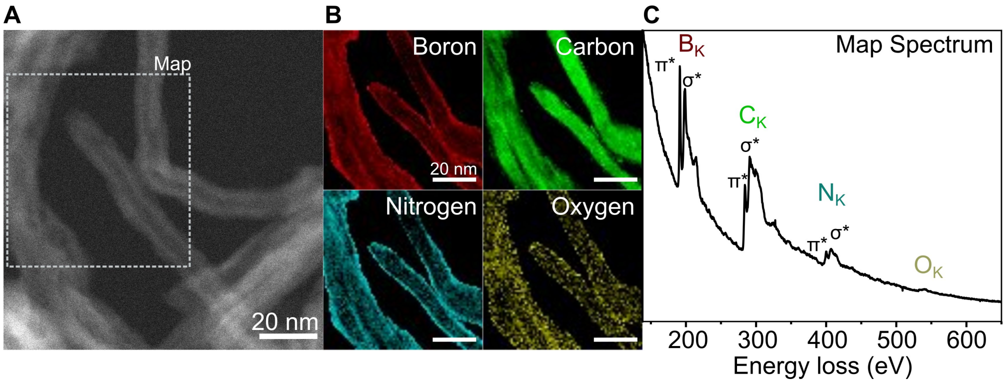

Figure S8: Example of MWCNT@BNNTs observed by (A) Dark Field STEM and (B) EELS spectral imaging, which reveals a BN shell around a C core. Elements detected, including oxygen, are summarised in (C) the correspond EELS map spectrum. Fine edge structures of B/N and C exhibit π^* and σ^* features typical of h-BN and graphitic C.

Table S3: Quantitative analysis of EELS spectral images. Atomic ratios are presented relative to carbon areal density. Percent contents are presented based on absolute areal density comparisons.

	**Atomic Ratio (/C)**				**Percent Content %**			
**Spectral Images**	B	C	N	O	B	C	N	O
Fig. S8	0.44 ± 0.063	1.00	0.39 ± 0.055	0.13 ± 0.019	22.61	50.8	19.74	6.85
Fig. S7	0.38 ± 0.054	1.00	0.35 ± 0.050	0.10 ± 0.014	20.9	54.48	19.3	5.32
Fig. 2D	0.35 ± 0.050	1.00	0.32 ± 0.046	0.10 ± 0.014	19.77	56.38	18.25	5.60

X-ray Photoelectron Spectroscopy

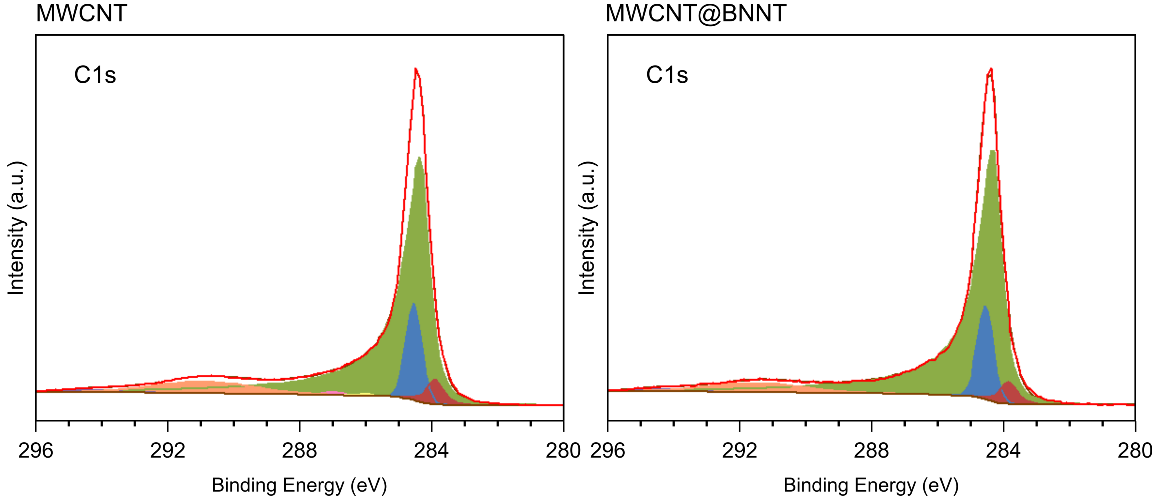

Figure S9: XPS High Resolution C1s spectra of MWCNTs and MWCNT@BNNTs, deconvoluted according to the data shown in Table S4 below. The two spectra are very similar, with no obvious change in the C1s of the MWCNT@BNNT to suggest significant covalent interactions between the MWCNT core and h-BN shell. Oxidised carbon species appear to be reduced in the MWCNT@BNNT. However oxygen in still present in the MWCNT@BNNT bonded to the h-BN, as evidenced in the B1s and N1s spectra of Fig. 3.

Table S4: Summarised description of synthetic peaks fitted to the high resolution XPS C1s spectrum of Nanocyl NC7000 MWCNTs and MWCNT@BNNTs as shown in Figure S9 above. Line shapes for peaks are Gaussian-Lorentzian, with the exception of the asymmetric modified Lorentzian LF lineshape for fitting sp^2 carbon due to its XPS final state effects.[12] Bond identification is referenced from[12] and.[13]

**MWCNT**				**MWCNT@BNNT**			
**Bond**	**Binding Energy (eV)**	**FWHM**	**% Area**	**Bond**	**Binding Energy (eV)**	**FWHM**	**% Area**
Defective carbon	283.9	0.60	3.56	Defective carbon	283.9	0.60	3.38
sp^2 carbon	284.4	0.65	74.26	sp^2 carbon	284.3	0.56	77.41
Disordered carbon	284.6	0.56	11.88	Disordered carbon	284.6	0.56	11.89
C-O	286.0	0.6	0.24	C-O	286.0	0.2	0.00
C=O	287.0	0.75	0.50	C=O	287.0	0.5	0.01
O-C=O	288.0	0.75	0.25	O-C=O	288.0	0.51	0.00
π-π*	290.9	3.37	8.61	π-π*	291.5	3.27	6.64
Plasmon loss	294.4	1.68	0.72	Plasmon loss	294.5	1.51	0.57

Table S5: Summarised description of synthetic peaks fitted to the high resolution XPS B1s and N1s spectrum of MWCNT@BNNTs as shown in Fig.3G,H. High resolution peaks were fitted in CasaXPS with a GL-T lineshape for the B-N bonds[14] and GL lineshapes for other bonds. Bond identifications are referenced from.[15]

**B1s**				**N1s**			
**Bond**	**Binding Energy (eV)**	**FWHM (eV)**	**% Area**	**Bond**	**Binding Energy (eV)**	**FWHM (eV)**	**% Area**
B-N	190.49	1.15	73.01	N-B	398.10	1.10	74.91
$B-N_2O$	191.71	1.5	17.47	N-B-O/ N-N	399.00	2.5	17.48
$B-NO_2$	192.76	1.61	9.52	N-O	401.00	1.97	3.23
				π-π*	406.35	3.23	4.38

Table S6: Summarised description of synthetic peaks fitted to the high resolution XPS B1s and N1s spectrum of BNNTs as shown in Fig.3I,J. High resolution peaks were fitted in CasaXPS with a GL-T lineshape for the B-N bonds and GL lineshapes for other bonds. Bond identifications are referenced from.[15]–[19]

**B1s**				**N1s**			
**Bond**	**Binding Energy (eV)**	**FWHM (eV)**	**% Area**	**Bond**	**Binding Energy (eV)**	**FWHM (eV)**	**% Area**
Defective B-N	189.67	1.58	19.65	Defective N-B	397.26	1.45	29.35
B-N	190.52	1.40	37.04	N-B	398.00	1.30	39.55
$B-N_2O$	191.7	1.55	20.74	N-B-O/ N-N	398.97	1.69	22.23
$B-NO_2$	192.78	1.60	12.85	N-O	400.40	1.98	6.71
BxOy	193.84	2.00	9.73	π-π*	406.57	2.91	2.15

UV-Vis Spectroscopy

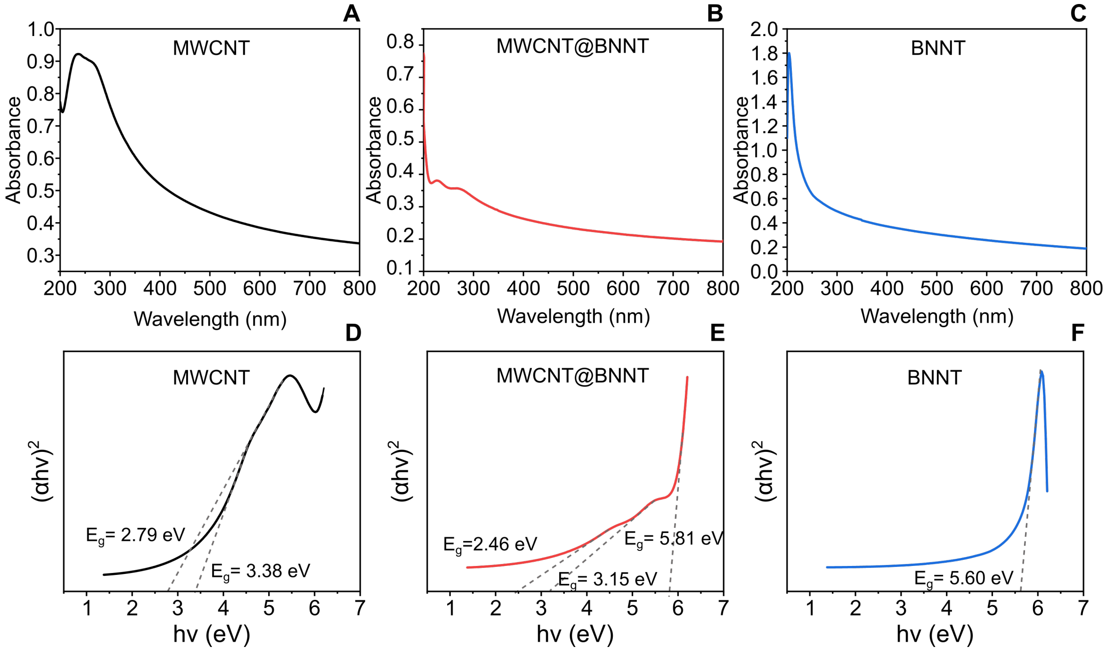

Figure S10: (A-C) UV-Vis absorption spectra of raw Nanocyl NC7000 MWCNT, and synthesised MWCNT@BNNT and BNNTs. (D-F) Corresponding Tauc plot with extrapolated optical band gap values shown. Given that the multiple different band gaps extrapolated from the Tauc plot of MWCNT@BNNT can be individually matched to both the MWCNT and h-BN components, the van der Waals hybridisation of these nanotubes is further corroborated.

Thermogravimetric Analysis TGA

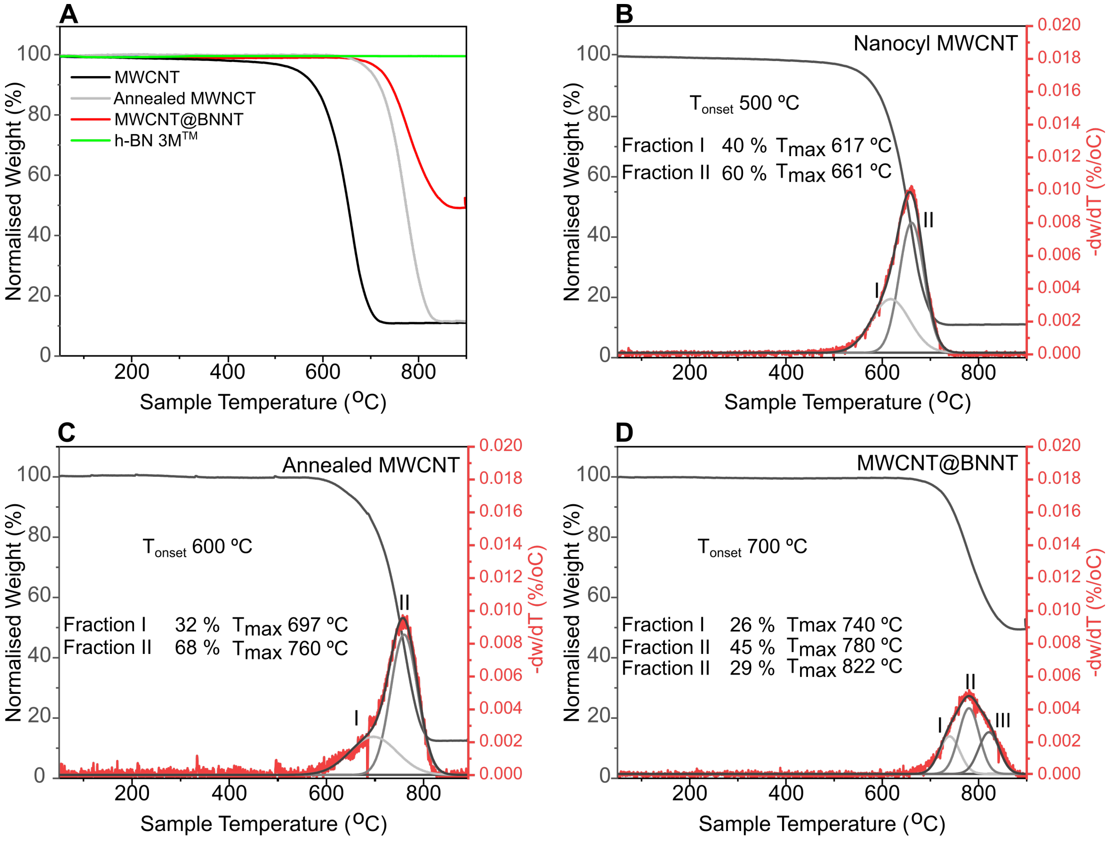

Figure S11: TGA graphs showing normalised weight % vs. temperature for Nanocyl MWCNTs, annealed MWCNTs and MWCNT@BNNTs compared to h-BN reference (3M™powder). TGA scan rate: 20 °C/min. (B-D) Individual TGA curves for MWCNT, annealed MWCNT and MWCNT@BNNTs are shown alongside their 1st-order derivative(wt%/°C) graphs dw/dT. Gaussian distributions are fitted to the derivative curves to identify oxidation fractions, their temperatures at maximum combustion rate (T_{max}) and composition contribution (area %). Temperature at oxidation onset (T_{onset}) is also shown.

FTIR

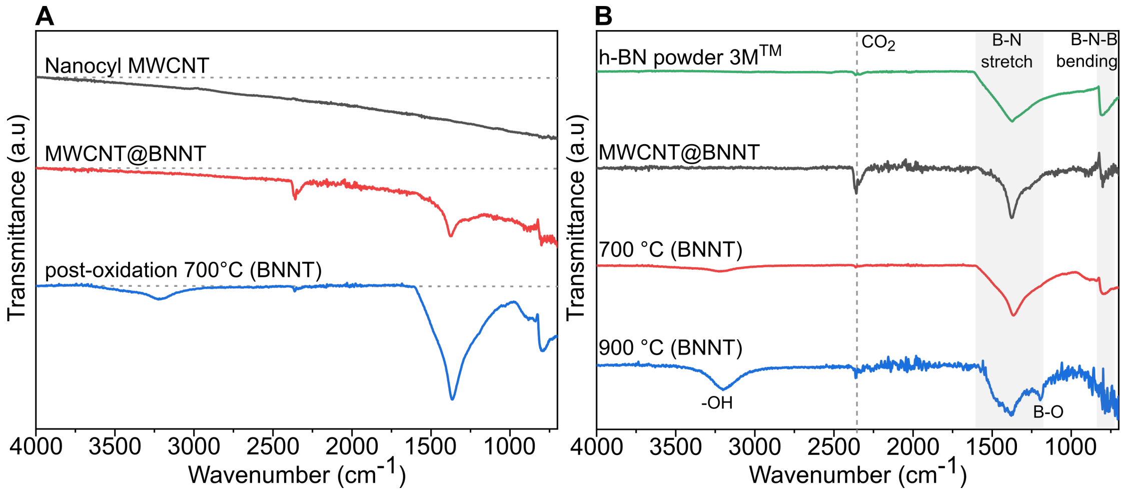

Figure S12: FTIR transmission spectra (A) as-collected from Nanocyl MWCNTs, MWCNT@BNNTs and BNNTs (made by 700°C oxidation of MWCNT@BNNT templates).The featureless MWCNT spectrum is in contrast to the MWCNT@BNNT and BNNT spectra, which both show absorption peaks associated with h-BN. (B) Normalised FTIR spectra comparing BNNTs (synthesised from 700°C and 900°C sacrificial templating) to MWCNT@BNNT (baseline substracted) and an h-BN reference (3M™ powder).

Table S7: Summary of FTIR absorption peaks recorded from spectra of MWCNT@BNNT, BNNT (700 °C), BNNT (900 °C) and reference h-BN powder from 3M™ (shown in Figure S12B). Note that the peak at ca. 2360 cm^{-1} is a result of atmospheric and/or adsorbed CO_2.

**FTIR Frequencies (cm^{-1})**					
**h-BN 3M™**	**MWCNT@BNNT**	**BNNT (700 °C)**	**BNNT (900 °C)**	**Vibration**	**Molecule/ Functional group**
-	-	3217	3196	O-H stretching	H_2O; B-OH
2362	2359	2361	2360	C-O asymmetric stretching	CO_2
1373	1377	1363	1380	B-N in-plane stretching	h-BN
-	-	-	1187	B-O stretching	B-O stretching
804	798	787	-	B-N-B out-of-plane bending	h-BN

Raman Spectroscopy

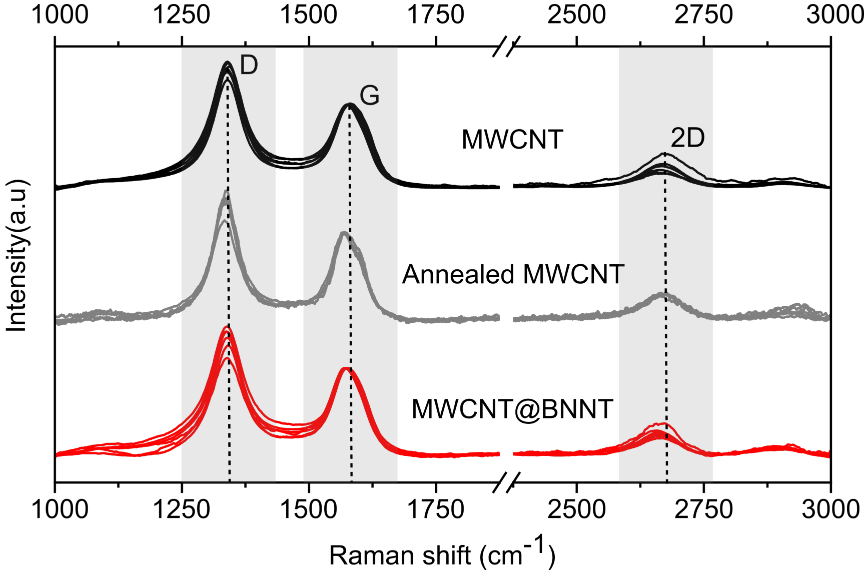

Figure S13: Raman spectra of Nanocyl NC7000™ MWCNTs, annealed MWCNTs (to 1000 °C in Ar/H_2), and MWNCT@BNNT. Six spectra were taken from each sample using a 532 nm wavelength laser. D, G and 2D band positions are highlighted. See Table S8 below for spectra analysis. If B and N had become primarily covalently incorporated into the graphitic lattice of the MWCNT, an increase in defect density would be detected in the Raman spectra of the MWCNT@BNNT as an increase in the I_D/I_G ratio relative to that of the pristine MWCNTs. However, I_D/I_G of MWCNT@BNNT Raman spectra do not exhibit such an increase, thereby substantiating that this synthesis technique favours h-BN deposition as a shell around the MWCNT core, the latter which maintains it graphitic lattice structural integrity.

Table S8: Measured average D,G and 2D Raman peak parameters (Raman shift, FWHM) and intensity ratios of I_D/I_G and I_{2D}/I_G for MWCNTs, Annealed MWCNTs and MWCNT@BN. Errors are standard deviations.

	**D peak**		**G peak**		**2D peak**	
	**Position (cm^{-1})**	**FWHM (cm^{-1})**	**Position (cm^{-1})**	**FWHM (cm^{-1})**	**Position (cm^{-1})**	**FWHM (cm^{-1})**
MWCNT	1340±1	80±6	1578±4	71±3	2666±5	111±13
Annealed MWCNT	1335±2	60±6	1569±2	62±4	2671±6	100±8
MWCNT@BNNT	1339±1	80±10	1574±2	69±4	2664±6	98±5

	**Peak ratios**	
	**I_D/I_G**	**I_{2D}/I_G**
MWCNT	1.43±0.08	0.25±0.09
Annealed MWCNT	1.35±0.11	0.29±0.02
MWCNT@BNNT	1.33±0.12	0.27±0.06

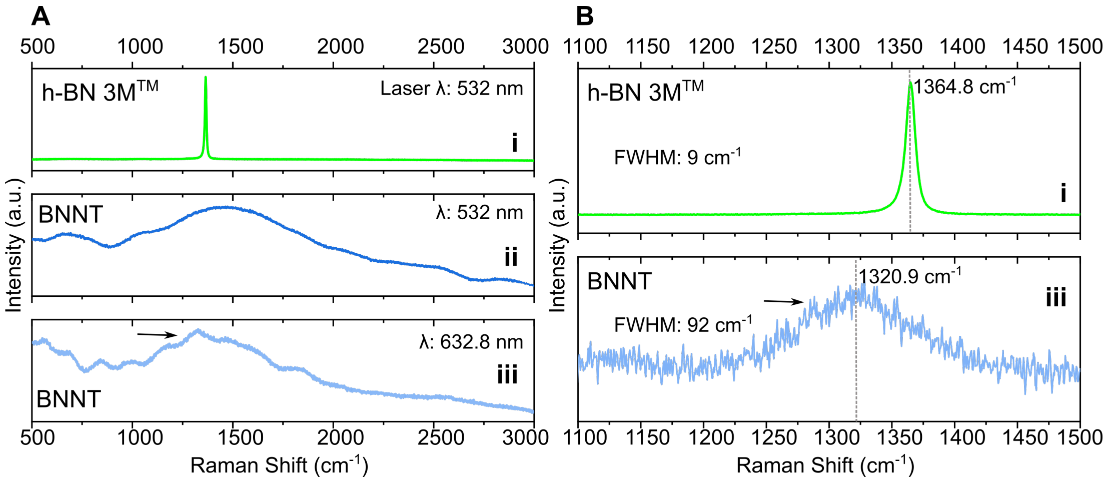

Figure S14: (A) Raman spectra collected from (i) a reference crystalline commercial 3M™ h-BN sample and (ii-iii) BNNTs synthesised by sacrificial templating. Laser wavelength used for (i-ii) was 532 nm, but 632.8 nm for (iii) to test fluorescence suppression. [20] (B) A magnification of the E_{2g} mode for (i) reference h-BN powder and (iii) the BNNTs (laser λ 632.8 nm) for clearer comparison of peak position and FWHM. FWHM are calculated from Lorentz peak fittings. Besides fluorescence suppression, the BNNT Raman spectra, and particulary the E_{2g} mode, is also likely influenced by anharmonic effects such as temperature dependence. For example, the generally broader E_{2g} peak of BNNTs relatve to bulk h-BN may be due to laser heating induced anharmonicity. [21],[22] Anharmonic thermal effects by laser heating may also cause the E_{2g} peak of BNNTs to downshift in frequency. [22],[23] Without accounting for such effects, and according to Nemanich *et al.* [24] a smaller crystalline size (L_a) of h-BN results in broader FWHM of the E_{2g} mode and an up-shift in peak frequency.

Measurement of MWCNT and MWCNT@BNNT Buckypaper Electrical Resistivity

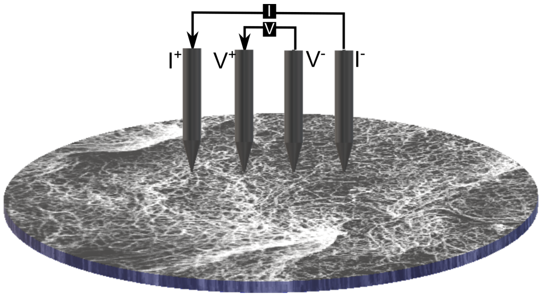

Figure S15: Illustration of Four Point Probe setup

A four point probe was used to measure the resistivity of the MWCNT and MWCNT@BNNT buckypapers.

The four point probe has two inner probes that measure voltage (V) and two outer probes that supply current (I), as shown in schematic in Figure SS15. A 1 μA current was applied. Output voltage (V) readings were taken for 10 positions on each buckypaper, including on both sides and after rotations of the buckypaper. The calibration of the voltage readings was checked with ITO glass. The buckypaper resistivity(ρ_o), was calculated according to the equation below for resistivity of thin film samples which have a thickness much less than the distance between the probes (i.e. t≪S):

$$\rho_o = \frac{\pi}{ln2}\left(\frac{V}{t}\right) I \tag{1}$$

Standard deviations were calculated for measured values of V (i.e. ΔV) and t (i.e. Δt). The error on resistivity $\Delta\rho_o$ was propagated as follows:

$$constant(c) = \frac{\pi}{ln2} I \tag{2}$$

$$\therefore \rho_o = c \cdot \left(\frac{V}{t}\right) \tag{3}$$

$$\Delta\rho_o = c \cdot \left(\frac{V}{t}\right) \sqrt{\left(\frac{\Delta V}{V}\right)^2 + \left(\frac{\Delta t}{t}\right)^2} \tag{4}$$

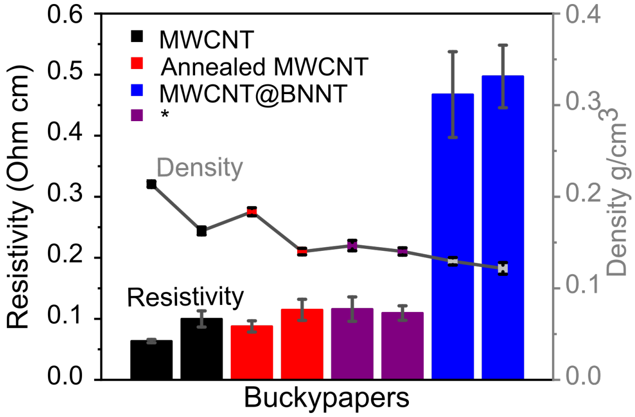

Figure S16: Electrical resistivity measurements of buckypapers reveal an increased resistivity of the MWCNT@BNNT buckypaper relative to the MWCNT, Annealed MWCNT and * buckypapers (Annealed: treated to 1000°C in inert atmosphere as a control). The * buckypaper was manufactured by coating a pre-fabricated MWCNT buckypaper with h-BN instead of fabricating a buckypaper with MWCNT@BNNT powder. Buckypaper density variation is not considered to be a contributing factor to the noticeable MWCNT@BNNT buckypaper resistivity increase.

Scanning Thermal Microscopy Setup

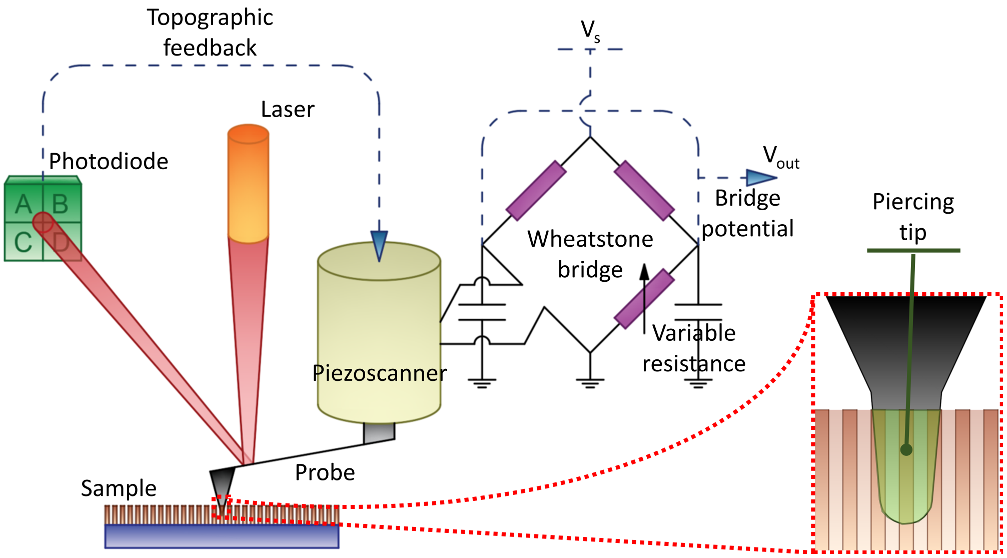

Figure S17: Schematics of the SThM setup. The Wheatstone bridge operates at AC frequency of 91 kHz to reduce the 1/f noise of the probe, with the probe power provided both by the AC voltage and DC offset.

Electronics for SThM signal processing

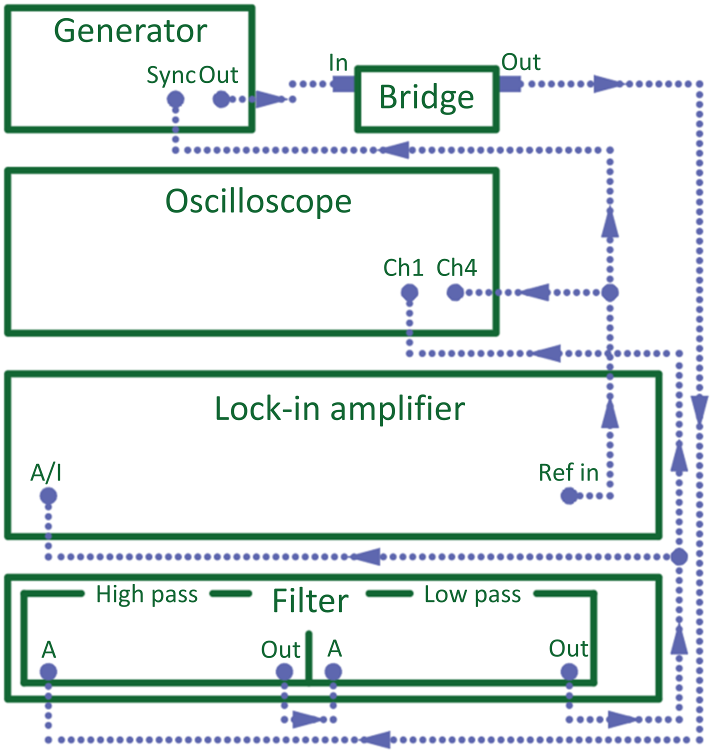

Figure S18: Schematics of the connections of the electronics for SThM signal processing. The Generator produces a combined AC+DC voltage in the range of 1-3V, Wheatstone bridge reduces the driving voltage in the ratio of 1/5 and is balanced before the measurements to provide zero AC output. Wheatstone bridge output that is preamplified by the instrumental amplifier and the band pass Filter adjusted to the 91 kHz +/- 2 kHz. The output of the Filter is detected by the Lock-in amplifier that detects the AC component proportional to the temperature of the probe.[25],[26]

References

1 Kim, K. S.; Kingston, C. T.; Hrdina, A.; Jakubinek, M. B.; Guan, J.; Plunkett, M.; Simard, B. Hydrogen-catalyzed, pilot-scale production of small-diameter boron nitride nanotubes and their macroscopic assemblies. *ACS Nano* **2014**, *8*, 6211–6220.

2 Jakubinek, M. B.; Niven, J. F.; Johnson, M. B.; Ashrafi, B.; Kim, K. S.; Simard, B.; White, M. A. Thermal conductivity of bulk boron nitride nanotube sheets and their epoxy-impregnated composites. *physica status solidi (a)* **2016**, *213*, 2237–2242.

3 Kim, K. S.; Jakubinek, M. B.; Martinez-Rubi, Y.; Ashrafi, B.; Guan, J.; O'neill, K.; Plunkett, M.; Hrdina, A.; Lin, S.; Dénommée, S.; Kingston, C.; Simard, B. Polymer nanocomposites from free-standing, macroscopic boron nitride nanotube assemblies. *RSC Advances* **2015**, *5*, 41186–41192.

4 Ban, C.; Jiang, X.; Li, L.; Liu, X. The piezoelectric and dielectric properties of flexible, nanoporous, self-assembled boron nitride nanotube thin films. *Journal of Materials Science* 1–11.

5 Nautiyal, P.; Zhang, C.; Loganathan, A.; Boesl, B.; Agarwal, A. High Temperature Mechanics of Boron Nanotube "Buckypaper" for Engineering Advanced Structural Materials. *ACS Applied Nano Materials* **2019**,

6 Kang, J. H.; Sauti, G.; Park, C.; Yamakov, V. I.; Wise, K. E.; Lowther, S. E.; Fay, C. C.; Thibeault, S. A.; Bryant, R. G. Multifunctional electroactive nanocomposites based on piezoelectric boron nitride nanotubes. *ACS Nano* **2015**, *9*, 11942–11950.

7 Adnan, M.; Marincel, D. M.; Kleinerman, O.; Chu, S.-H.; Park, C.; Hocker, S. J.; Fay, C.; Arepalli, S.; Talmon, Y.; Pasquali, M. Extraction of Boron Nitride Nanotubes and Fabrication of Macroscopic Articles Using Chlorosulfonic Acid. *Nano Letters* **2018**, *18*, 1615–1619.

8 Chang, C.-W.; Han, W.-Q.; Zettl, A. Thermal conductivity of BCN and BN nanotubes. *Journal of Vacuum Science & Technology B: Microelectronics and Nanometer Structures Processing, Measurement, and Phenomena* **2005**, *23*, 1883–1886.

9 Tang, C.; Bando, Y.; Liu, C.; Fan, S.; Zhang, J.; Ding, X.; Golberg, D. Thermal conductivity of nanostructured boron nitride materials. *The Journal of Physical Chemistry B* **2006**, *110*, 10354–10357.

10 Hong, X.; Wang, D.; Chung, D. Boron nitride nanotube mat as a low-k dielectric material with relative dielectric constant ranging from 1.0 to 1.1. *Journal of Electronic Materials* **2016**, *45*, 453–461.

11 Hong, X.; Wang, D.; Chung, D. Strong viscous behavior discovered in nanotube mats, as observed in boron nitride nanotube mats. *Composites Part B: Engineering* **2016**, *91*, 56–64.

12 Blume, R.; Rosenthal, D.; Tessonnier, J.-P.; Li, H.; Knop-Gericke, A.; Schlögl, R. Characterizing Graphitic Carbon with X-ray Photoelectron Spectroscopy: A Step-by-Step Approach. *ChemCatChem* **2015**, *7*, 2871–2881.

13 Zielke, U.; Hüttinger, K.; Hoffman, W. Surface-oxidized carbon fibers: I. Surface structure and chemistry. *Carbon* **1996**, *34*, 983 – 998.

14 Jakubinek, M. B.; Kim, K. S.; Homenick, C.; Kodra, O.; Walker, S.; Simard, B. Assessment of boron nitride nanotube materials using X-ray photoelectron spectroscopy. *Canadian Journal of Chemistry* **2019**, *97*, 457–464.

15 Sediri, H.; Pierucci, D.; Hajlaoui, M.; Henck, H.; Patriarche, G.; Dappe, Y. J.; Yuan, S.; Toury, B.; Belkhou, R.; Silly, M. G.; Sirotti, F.; Boutchich, M.; Ouerghi, A. Atomically Sharp Interface in an h-BN-epitaxial graphene van der Waals Heterostructure. *Scientific Reports* **2015**, *5*, 1–10.

16 Guimon, C.; Gonbeau, D.; Pfister-Guillouzo, G.; Dugne, O.; Guette, A.; Naslain, R.; Lahaye, M. XPS study of BN thin films deposited by CVD on SiC plane substrates. *Surface and Interface Analysis* **1990**, *16*, 440–445.

17 Bresnehan, M. S.; Hollander, M. J.; Wetherington, M.; Wang, K.; Miyagi, T.; Pastir, G.; Snyder, D. W.; Gengler, J. J.; Voevodin, A. A.; Mitchel, W. C.; Robinson, J. A. Prospects of direct growth boron nitride films as substrates for graphene electronics. *Journal of Materials Research* **2014**, *29*, 459–471.

18 Wagner, C.; Riggs, W.; Davis, L.; Moulder, J.; Muilenberg, G. Handbook of X-ray photoelectron spectroscopy, Perkin-Elmer Corp. *Eden Prairie, MN* **1979**, *38*.

19 Sutter, P.; Lahiri, J.; Zahl, P.; Wang, B.; Sutter, E. Scalable synthesis of uniform few-layer hexagonal boron nitride dielectric films. *Nano Letters* **2013**, *13*, 276–281.

20 Ferraro, J. R.; Nakamoto, K.; Brown, C. W. *Introductory Raman Spectroscopy.*, 2nd ed.; Ebook central; 2003.

21 Arenal, R.; Ferrari, A. C.; Reich, S.; Wirtz, L.; Mevellec, J.-Y.; Lefrant, S.; Rubio, A.; Loiseau, A. Raman Spectroscopy of Single-Wall Boron Nitride Nanotubes. *Nano Letters* **2006**, *6*, 1812–1816.

22 Arutyunyan, N. R.; Obraztsova, E. D.; Silly, M.; Jaffrennou, P.; Attal-Tretout, B.; Loiseau, A.; Chuvilin, A. L. Thermal effects in Raman spectra of hexagonal boron nitride and nanotube-containing boron nitride soot. *physica status solidi (b)* **2006**, *243*, 3316–3319.

23 Lu, J.; Ren, Q.; Sun, L.; Yu, J.; Chen, Y.; Shen, X.; Chen, Z. Temperature-dependent Raman spectra of bamboo-like boron nitride nanotubes. *Applied Physics Express* **2014**, *7*, 022401.

24 Nemanich, R.; Solin, S.; Martin, R. M. Light scattering study of boron nitride microcrystals. *Physical Review B* **1981**, *23*, 6348.

25 Spiece, J.; Evangeli, C.; Lulla, K.; Robson, A.; Robinson, B.; Kolosov, O. Improving accuracy of nanothermal measurements via spatially distributed scanning thermal microscope probes. *Journal of Applied Physics* **2018**, *124*, 015101.

26 Tovee, P.; Pumarol, M.; Zeze, D.; Kjoller, K.; Kolosov, O. Nanoscale spatial resolution probes for scanning thermal microscopy of solid state materials. *Journal of Applied Physics* **2012**, *112*, 114317.
